# Self-harm presentation across healthcare settings by sex in young people: an e-cohort study using routinely collected linked healthcare data in Wales, UK

**DOI:** 10.1136/archdischild-2019-317248

**Published:** 2019-10-14

**Authors:** Amanda Marchant, Samantha Turner, Lloyd Balbuena, Evyn Peters, Dave Williams, Keith Lloyd, Ronan Lyons, Ann John

**Affiliations:** 1 Swansea University Medical School, Swansea University, Swansea, UK; 2 Department of Psychiatry, University of Saskatchewan, Saskatoon, Saskatchewan, Canada; 3 Child and Adolescent Psychiatry, Aneurin Bevan Health Board, Newport, UK

**Keywords:** Child Psychiatry, Accident & Emergency, Adolescent Health, Epidemiology, Health Service

## Abstract

**Background:**

This study used individual-level linked data across general practice, emergency departments (EDs), outpatients and hospital admissions to examine contacts across settings and time by sex for self-harm in individuals aged 10–24 years old in Wales, UK.

**Methods:**

A whole population-based e-cohort study of routinely collected healthcare data was conducted. Rates of self-harm across settings over time by sex were examined. Individuals were categorised based on the service(s) to which they presented.

**Results:**

A total of 937 697 individuals aged 10–24 years contributed 5 369 794 person years of data from 1 January 2003 to 30 September 2015. Self-harm incidence was highest in primary care but remained stable over time (incident rate ratio (IRR)=1.0; 95% CI 0.9 to 1.1). Incidence of ED attendance increased over time (IRR=1.3; 95% CI 1.2 to 1.5) as did hospital admissions (IRR=1.4; 95% CI 1.1 to 1.6). Incidence in the 15–19 years age group was the highest across all settings. The largest increases were seen in the youngest age group. There were increases in ED attendances for both sexes; however, females are more likely than males to be admitted following this. This was most evident in individuals 10–15 years old, where 76% of females were admitted compared with just 49% of males. The majority of associated outpatient appointments were under a mental health specialty.

**Conclusions:**

This is the first study to compare self-harm in people aged 10–24 years across primary care, EDs and hospital settings in the UK. The high rates of self-harm in primary care and for young men in EDs highlight these as important settings for intervention.

**What is already known?**

- Most self-harm research is conducted in hospital settings although many individuals are managed in primary care.
- There has been no previous research examining linked whole population GP and hospital admissions nor incorporating emergency department data.
- Preferential service presentation by age and sex in linked data is unknown.

**What this study adds?**

- This is the first study of its kind in the UK to examine contacts for self-harm across GP, hospital admissions, outpatients and emergency departments.
- Admissions and emergency department attendances increased for those aged 10–14, and emergency department attendances increased for those aged 15–19 years old.
- Males were less likely to be admitted to hospital following emergency department attendance for self-harm even in the case of self-poisoning and in under 16s.

## Background

Self-harm (SH) refers to any act of intentional self-injury or poisoning regardless of suicidal intent or motivation.^1 2^ Accurate data on SH are crucial to suicide prevention efforts. A history of SH is one of the strongest risk factors associated with subsequent suicide.^3^ Up to 80% of suicide decedents have a primary or secondary care mental health contact in the year before death.^4^ These contacts present an opportunity for intervention. Approximately 25% of women and 10% of men aged 16–24 reported having self-harmed in the past.^5^ Data underpinning policy and practice are often derived from populations attending emergency departments (EDs) or admitted to hospital.^6–8^

Approximately twice as many people who self-harm seek help in primary care than access secondary care,^9^ and many are managed in primary care.^10 11^ There have been routinely collected data studies based solely in this setting.^9 12^ A significant increase in the incidence of SH in young adolescent girls was found from 2011 to 2014 combining hospital admissions and general practice (GP) data, with no corresponding increase in older adolescents or males.^11^ Little is known about how SH contacts differ across settings at the population level; for example, are the same people attending GPs and EDs?

The Secure Anonymised Information Linkage (SAIL) databank^13 14^ presents a unique opportunity to link data from primary care, EDs and hospitals at the person level to explore contacts for SH. This level of linkage across services at the population level is not possible in any other routinely collected healthcare data source currently held in the UK. Using such data to examine presentation across services can inform policy and practice, identifying opportunities for intervention. The aims of the current study are to examine incidence over time of SH in a whole population of young people across primary care, ED, hospital outpatients and admissions, and to explore if individuals preferentially present across different settings.

## Methods

### Study design

This is a retrospective e-cohort study.

### Data source

The SAIL databank (www.saildatabank.com) is an expanding data repository of anonymised person-based linkable data from healthcare and public settings to support research (online supplementary appendix A). Policies and procedures have been described in detail previously.^13 14^

10.1136/archdischild-2019-317248.supp1 Supplementary data

### Study population and setting

We used multiple data sets linked at the patient level: Welsh Demographic Service; Welsh Index of Multiple Deprivation, containing deprivation scores for all lower super output areas in Wales^15^; GP database, containing information for all GP interactions covering 79% of the Welsh population (70% considered acceptable in prevalence studies^16^); ED data set, containing data for all NHS Wales ED attendances (34 sites, including minor injuries units); Patient Episode Database for Wales, containing data for all NHS Wales hospital admissions; outpatients data; and Office for National Statistics death register. For further details of these data sets, see online supplementary appendix A or www.saildatabank.com.

Individuals aged 10–24 registered with a SAIL supplying GP from 1 January 2003 to 30 September 2015 were selected as the baseline population to give a common population denominator allowing comparisons across settings. Data collection began 1 year after GP registration, 10th birthday or study onset, whichever was the latest. Data collection ended on the date of GP deregistration, death, 25th birthday or study end, whichever was sooner. Individuals could supply multiple data periods.

### Measures

Age and deprivation indices were collected based on the data collection onset each year. Children and young people (CYP) are defined as those aged 10–24.^17^ Age was categorised into three groups of equal age bands, 10–14, 15–19 and 20–24 years, in keeping with previous research.^18 19^ The National Institute for Health and Care Excellence (NICE) guidance recommends admission for under 16s attending EDs with SH.^1^ We explored this where relevant by dividing into age bands 10–15, 16–18 and 19–24 years.

Measures were taken from GP data using validated primary care Read codes.^9 12^ Additional Read codes were identified through manual searching and checked by a clinician (AJ, KL; online supplementary appendix B). Hospital admissions for SH were identified based on the International Classification of Diseases 10th revision (ICD-10) codes for SH (X60-X84) and undetermined intent (Y10-Y34). A standard coding system is employed across EDs in Wales, grouping by attendance type and diagnosis. While this does not contain the diagnostic detail of ICD-10 and Read codes, this is sufficient for identifying SH. SH was defined as an attendance recorded as ‘deliberate SH’.^20^ Additional analysis was conducted to identify appropriate diagnostic codes when an event of undetermined intent was recorded (online supplementary appendix C). Codes referring to SH with alcohol were excluded from all data sets unless they were recorded alongside another relevant code.^21^

10.1136/archdischild-2019-317248.supp2 Supplementary data

10.1136/archdischild-2019-317248.supp3 Supplementary data

#### Trends over time

Incidence was defined as no SH record within the previous 12 months.^22–24^ ED data are available from August 2009. A full year of history is required to allow the necessary 1 year to distinguish an incident from a prevalent case. As such ED data are presented from 2011 to 2015 only, with 2010 used only to assess whether an individual has attended previously (denominators adjusted).

SH method over time (2003–2015) was examined. All GP events and hospital admissions with a record of SH were included (as opposed to annual incidence described above). Method was broken down into ‘self-poisoning’ and ‘self-injury’.

#### Contacts across services

Presentations to each service for SH from 1 August 2009 to 30 September 2015 were identified. This is the maximum period where data coverage is available across all settings (online supplementary figure 1). Data sets were linked at the level of the individual. Participants and SH events were divided into mutually exclusive groups based on the service(s) to which they presented (eg, GP only, GP and ED, and so on). Age and deprivation data were taken from the first SH presentation during this time. An SH event was defined as a record of SH in one or more service on a given date. SH method was examined. Each participant could have multiple events across services. We examined admission specialty. This is the specialty under which the patient was treated, and is either the consultant’s main specialty or a different specialty function which is the consultant’s interest specialty function. These specialties were broadly grouped into the following categories: ‘surgical specialties’, which included all surgical specialties (eg, general, plastic and so on), with the exception of paediatric surgery which was categorised under ‘paediatrics’ and ‘Accident and Emergency surgical specialty’ which was examined separately; ‘Accident and Emergency surgical specialty’; ‘paediatric specialties’, encompassing all paediatric specialties with the exception of child and adolescent psychiatry; ‘psychiatric’, which consists of all psychiatric specialties (eg, psychiatric intensive care, eating disorders and so on, including child and adolescent psychiatry); ‘general medicine’; and ‘other’, encompassing all other medical specialties (see online supplementary appendix D for a full breakdown of specialty codes).

10.1136/archdischild-2019-317248.supp4 Supplementary data

10.1136/archdischild-2019-317248.supp5 Supplementary data

Admission to hospital within 7 days of ED attendance was examined as a measure of whether an ED attendance resulted in admission. A 7-day window allows for an individual to remain in ED prior to admission and for delays in data recording. Whether an individual was seen in outpatients and by which specialty in the 30 days following SH was examined.

### Statistical analysis

The SAIL databank was interrogated using structured query language.

Annual incidence rates were calculated using person years at risk (PYAR) as a denominator. PYAR is a more appropriate unit than the number of registered cases because each individual’s follow-up period is not fixed.^25^ Poisson regression was undertaken to investigate the adjusted association between incidence of SH in each data set (data sets examined individually) and year of diagnosis, sex, age group and deprivation. Poisson regression modelling was additionally used to assess interactions between demographic variables. Wald tests were used to assess significance of findings. Robust SEs for the estimated incident rate ratios (IRRs) were used to account for clustering within practices. Analysis was conducted in SPSS V.22.

## Results

### Study population

In total 937 697 individuals aged 10–24 provided 5 369 794 person years of data from 1 January 2003 to 30 September 2015 (online supplementary table 1).

10.1136/archdischild-2019-317248.supp6 Supplementary data

### Incidence of SH over time

Incidence of SH in primary care remained stable over time, while incidence of ED attendances and hospital admissions has increased (table 1).

**Table 1:** Number of events, incidence per 1000 PYAR (95% CI) and IRR* (95% CI)† for presentation to services for self-harm

Variable	GP event		Emergency department attendance‡		Hospital admission	
	Events (n); incidence	IRR	Events (n); incidence	IRR	Events (n); incidence	IRR
Gender						
Male	8506; 3.1 (3 to 3.2)	Reference (p<0.0001)	3387; 3.4 (3.3 to 3.5)	Reference (p<0.0001)	4665; 1.7 (1.6 to 1.7)	Reference (p<0.0001)
Female	16 345; 6.2 (6.2 to 6.3)	2.0 (1.9 to 2.1)	4161; 4.4 (4.3 to 4.5)	1.3 (1.2 to 1.4)	9304; 3.6 (3.5 to 3.6)	2.1 (2.0 to 2.3)
Age group						
10–14	4618; 2.5 (2.4 to 2.6)	Reference (p<0.0001)	1139; 1.8 (1.7 to 1.9)	Reference (p<0.0001)	2941; 1.6 (1.5 to 1.7)	Reference (p<0.0001)
15–19	11 782; 6.5 (6.4 to 6.7)	2.6 (2.4 to 2.8)	3578; 5.5 (5.3 to 5.7)	3.1 (2.8 to 3.6)	6526; 3.6 (3.5 to 3.7)	2.3 (2.1 to 2.5)
20–24	8451; 4.9 (4.8 to 5)	2.0 (1.8 to 2.2)	2831; 4.3 (4.2 to 4.5)	2.5 (2.1 to 2.9)	4502; 2.6 (2.5 to 2.7)	1.6 (1.5 to 1.8)
Deprivation§						
5	3026; 2.9 (2.8 to 3)	Reference (p<0.0001)	844; 2.2 (2.1 to 2.4)	Reference (p<0.0001)	1631; 1.6 (1.5 to 1.6)	Reference (p<0.0001)
4	3253; 3.6 (3.5 to 3.7)	1.3 (1.1 to 1.4)	867; 2.7 (2.5 to 2.9)	1.2 (1.1 to 1.4)	1872; 2.1 (2 to 2.2)	1.3 (1.2 to 1.5)
3	4198; 4.2 (4.1 to 4.3)	1.5 (1.3 to 1.6)	1239; 3.4 (3.2 to 3.6)	1.6 (1.4 to 1.8)	2386; 2.4 (2.3 to 2.5)	1.5 (1.4 to 1.7)
2	5329; 5.2 (5.1 to 5.4)	1.8 (1.7 to 2.0)	1726; 4.6 (4.4 to 4.9)	2.1 (1.8 to 2.4)	3091; 3 (2.9 to 3.1)	2.0 (1.8 to 2.2)
1	8225; 6.9 (6.8 to 7.1)	2.4 (2.2 to 2.6)	2692; 6.2 (6 to 6.4)	2.8 (2.5 to 3.2)	4550; 3.8 (3.7 to 3.9)	2.5 (2.2 to 2.7)
Year						
2003	1751; 4.5 (4.3 to 4.7)	Reference (p<0.0001)			846; 2.2 (2 to 2.3)	Reference (p<0.0001)
2004	1944; 4.6 (4.4 to 4.8)	1.0 (0.9 to 1.2)			954; 2.3 (2.1 to 2.4)	1.0 (0.9 to 1.2)
2005	2022; 4.7 (4.5 to 4.9)	1.0 (0.9 to 1.2)			1074; 2.5 (2.3 to 2.6)	1.1 (1.0 to 1.3)
2006	2018; 4.6 (4.4 to 4.8)	1.0 (0.9 to 1.1)			1172; 2.7 (2.5 to 2.8)	1.2 (1.1 to 1.4)
2007	2212; 5 (4.8 to 5.3)	1.1 (1.0 to 1.2)			1299; 3 (2.8 to 3.1)	1.3 (1.2 to 1.5)
2008	2241; 5.1 (4.9 to 5.3)	1.1 (1.0 to 1.2)			1222; 2.8 (2.6 to 2.9)	1.3 (1.1 to 1.4)
2009	1934; 4.4 (4.2 to 4.6)	1.0 (0.9 to 1.1)			1059; 2.4 (2.3 to 2.6)	1.1 (1.0 to 1.3)
2010	1988; 4.6 (4.4 to 4.8)	1.0 (0.9 to 1.1)			1007; 2.3 (2.2 to 2.5)	1.1 (0.9 to 1.2)
2011	1855; 4.4 (4.2 to 4.6)	1.0 (0.8 to 1.1)	1444; 3.4 (3.2 to 3.6)	Reference (p<0.0001)	1063; 2.5 (2.4 to 2.7)	1.1 (1.0 to 1.3)
2012	1887; 4.5 (4.3 to 4.7)	1.0 (0.9 to 1.1)	1631; 3.9 (3.7 to 4.1)	1.1 (1.0 to 1.3)	1052; 2.5 (2.4 to 2.7)	1.1 (1.0 to 1.3)
2013	1939; 4.7 (4.5 to 4.9)	1.0 (0.9 to 1.2)	1612; 3.9 (3.7 to 4.1)	1.2 (1.0 to 1.3)	1205; 2.9 (2.8 to 3.1)	1.3 (1.1 to 1.6)
2014	1726; 4.3 (4.1 to 4.5)	0.9 (0.8 to 1.0)	1568; 3.9 (3.7 to 4.1)	1.2 (1.0 to 1.3)	1137; 2.8 (2.7 to 3)	1.3 (1.1 to 1.5)
2015¶	1334; 4.5 (4.3 to 4.8)	1.0 (0.9 to 1.1)	1293; 4.4 (4.2 to 4.7)	1.3 (1.2 to 1.5)	879; 3 (2.8 to 3.2)	1.4 (1.1 to 1.6)

*Adjusted for calendar year, age and deprivation.

†Based on Wald test.

‡Emergency department data from 2011 onwards only.

§Deprivation: 1=most deprived; 5=least deprived.

¶Data collected in 2015 up until 30 September—denominator for incidence rate adjusted accordingly but actual counts may appear lower.

GP, general practice; IRR, incident rate ratio; PYAR, person years at risk.

### Incidence over time by sex and age

Incidence rates and IRRs over time varied by age group (10–14, 15–19 and 20–24 years), sex and setting (online supplementary tables 2 and 3).

#### Individuals aged 10–14 years old

**Figure 1:**
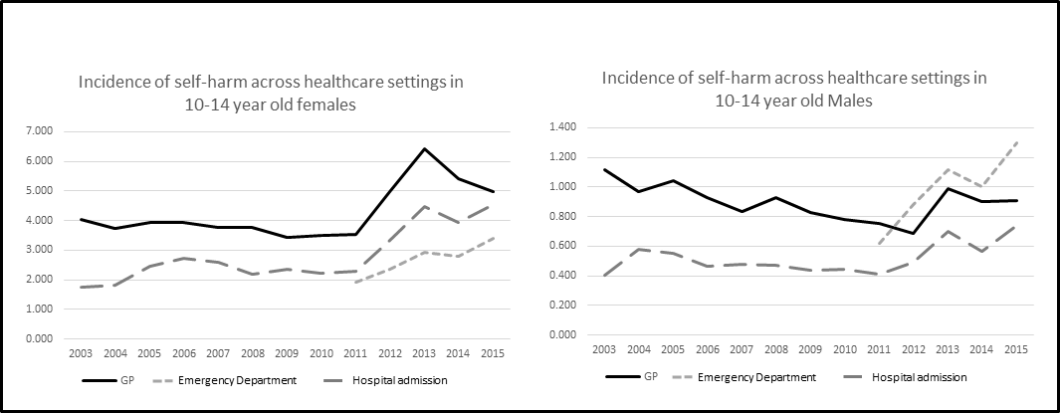
Incidence per 1000 PYAR of self-harm by service presentation and sex over time in individuals aged 10–14 years old. GP, general practice; PYAR, person years at risk.

Across services incidence was lowest in individuals aged 10–14 years old. Incidence over time increased for females across all settings. This was most marked from 2011 onwards (figure 1). For males there was no corresponding increase over time in GP attendances (females IRR=1.2 (95% CI 1.1 to 1.4; p<0.001); males IRR=0.8 (95% CI 0.6 to 1.1; p=0.136); interaction between sex and year IRR=1.1 (95% CI 1.0 to 1.1; p<0.001) per iteration for females, with males as the reference group). ED attendances increased significantly over time for both sexes (females IRR=1.8 (95% CI 1.4 to 2.3; p<0.001); males IRR=2.1 (95% CI 1.8 to 2.4; p<0.001); interaction between sex and year non-significant). Hospital admissions almost doubled over time for males, with an even larger increase over time for females (females IRR=2.6 (95% CI 2.2 to 3.0; p<0.001); males IRR=1.9 (95% CI 1.3 to 2.7; p=0.014); interaction between sex and year IRR=1.1 (95% CI 1.0 to 1.1; p=0.005)).

#### Individuals aged 15–19 years old

Incidence was highest in individuals aged 15–19 years old across all settings. There was no significant increase over time in GP contacts for either sex. ED attendances increased significantly for both sexes (females IRR=1.3 (95% CI 1.2 to 1.4; p<0.001); males IRR=1.3 (95% CI 1.1 to 1.5; p<0.001); interaction between sex and year non-significant). Hospital admissions increased significantly for females but not for males (females IRR=1.5 (95% CI 1.4 to 1.7; p<0.001); males IRR=1.0 (95% CI 0.8 to 1.2; p<0.001); interaction between sex and year IRR=1.0 (95% CI 1.0 to 1.0; p=0.001)).

#### Individuals aged 20–24 years old

There was no significant increase in GP attendance or hospital admissions over time for either sex. ED attendances increased significantly over time for females but not for males (females IRR=1.3 (95% CI 1.2 to 1.4; p<0.001); males IRR=1.0 (95% CI 0.9 to 1.2; p=0.007); interaction between sex and year non-significant).

### Changes in method over time by setting and sex

**Figure 2:**
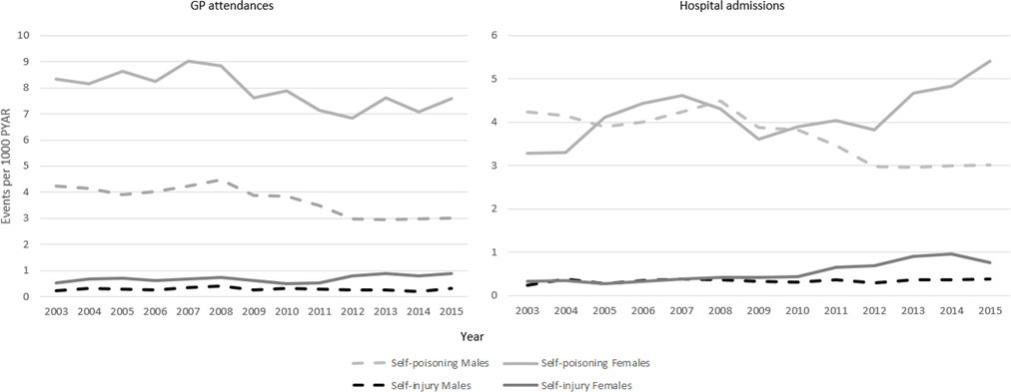
Self-harm events per 1000 PYAR by method, sex and setting over time. GP, general practice; PYAR, person years at risk.

GP attendances for self-poisoning decreased over time from 6.2 to 5.2 attendances per 1000 PYAR (IRR=0.8 (95% CI 0.7 to 1.0; p<0.001)). This decrease was seen for both sexes (interaction between sex and year non-significant; figure 2). In contrast hospital admissions for self-poisoning increased over time from 2.5 to 3.5 admissions per 1000 PYAR (overall IRR=1.4 (95% CI 1.1. to 1.7; p<0.001); males IRR=0.9 (95% CI 0.7 to 1.1; p<0.001); females IRR=1.6 (95% CI 1.4 to 2.0; p<0.001); interaction between sex and year IRR=1.0 (95% CI 1.0 to 1.1; p=0.016) per iteration, with males as the reference group).

GP attendances for self-injury increased significantly over time from 0.4 to 0.6 events per 1000 PYAR (overall IRR=1.6 (95% CI 1.2 to 2.1; p=0.039); males IRR=1.3 (95% CI 0.8 to 2.3; p<0.001); females IRR=1.7 (95% CI 2.3 to 2.3; p<0.001); interaction between sex and year non-significant). For GP attendances for self-injury, 89% (95% CI 88 to 90; n=2288) were for self-cutting. Hospital admissions for self-injury also increased over time from 2.5 to 3.5 admissions per 1000 PYAR (overall IRR=2.0 (95% CI 1.5 to 2.8; p<0.001)). There was no significant change over time for males (IRR=1.6 (95% CI 1.0 to 2.5; p=0.607)). Female admissions more than doubled over the study period (IRR=2.4 (95% CI 1.7 to 3.5; p<0.001); significant interaction between sex and year IRR=1.1 (95% CI 1.1 to 1.1)). Of hospital admissions for self-injury, 72% (95% CI 70 to 74; n=1649) were for self-cutting.

### SH incidence in relation to sex and deprivation

Across all settings incidence in the most deprived areas was more than double that in the least deprived areas. IRRs for deprivation are greater in males than in females, with incidence in the most deprived areas being more than three times that in the least deprived areas (IRRs GP male=3.1 (95% CI 2.8 to 3.3; p<0.001), female 2.1 (95% CI 2.0 to 2.3; p<0.001); ED male 3.5 (95% CI 3.1 to 3.8; p<0.001), female 2.4 (95% CI 2.2 to 2.7; p<0.001); hospital admissions male 3.2 (95% CI 2.9 to 3.6; p<0.001), female 2.2 (95% CI 2.2 to 2.4; p<0.001)) (online supplementary table 4). There was a significant interaction between sex and deprivation across all services, with incidence of SH in males increasing significantly more per deprivation fifth than females (GP IRR=1.1 (95% CI 1.1 to 1.2; p<0.001); ED IRR=1.1 (95% CI 1.1 to 1.2; p=0.002); hospital admissions IRR=1.1 (95% CI 1.1 to 1.2; p<0.001)).

### Contacts across services

Demographics of individuals presenting to each single service or to multiple services are shown in table 2. Individuals presenting to ‘GP only’ make up the largest group. Females outnumber males in every setting except ‘ED only’. Most events recorded in ‘GP only’ and ‘hospital admissions only’ were self-poisoning, while the majority of events recorded in ‘ED only’ were self-injury.

**Table 2:** Presentation of self-harm across services* and associated outpatient appointments from 1 August 2011 to 30 September 2015

	By individual†		By event‡						
	Individuals n (%; 95% CI)	Females n (%§; 95% CI)	Events n (%; 95% CI)	Self-poisoning only n (%; 95% CI)	Self-injury only n (%; 95% CI)	Self-poisoning and injury n (%; 95% CI)	Outpatients¶ n (%; 95% CI)	Mental health specialty n (%**; 95% CI)	Paediatric specialty n(%^††^; 95% CI)
GP only	3912 (25; 24 to 26)	2530 (65; 63 to 66)	10 690 (37; 36 to 37)	6194 (58; 57 to 59)	4181 (39; 38 to 40)	315 (3; 3 to 3)	2504 (23; 23 to 24)	1758 (70; 67 to 73)	90 (4; 3 to 4)
Hospital admission only	1084 (7; 7 to 7)	652 (60; 57 to 63)	4171 (14; 14 to 15)	3364 (81; 79 to 82)	673 (16; 15 to 17)	134 (3; 3 to 4)	1308 (31; 30 to 33)	907 (69; 65 to 74)	46 (4; 3 to 5)
ED only	3428 (22; 21 to 22)	1449 (42; 41 to 44)	7471 (26; 25 to 26)	1371 (18; 17 to 19)	5075 (68; 67 to 69)	1025 (14; 13 to 15)	1882 (25; 24 to 26)	1080 (57; 54 to 61)	46 (2; 2 to 3)
GP and hospital admission	2394 (15; 15 to 16)	1725 (72; 70 to 74)	2270 (8; 8 to 8)	1633 (72; 70 to 74)	149 (7; 6 to 8)	488 (21; 20 to 23)	734 (32; 30 to 35)	592 (81; 74 to 87)	28 (4; 3 to 5)
GP and ED	1979 (13; 12 to 13)	1150 (58; 56 to 60)	2144 (7; 7 to 8)	518 (24; 22 to 26)	474 (22; 20 to 24)	1152 (54; 52 to 56)	545 (25; 23 to 28)	408 (75; 67 to 82)	8 (1; 1 to 3)
ED and hospital admission	566 (4; 3 to 4)	331 (58; 54 to 62)	1133 (4; 4 to 4)	308 (27; 25 to 30)	157 (14; 12 to 16)	668 (59; 56 to 62)	413 (36; 33 to 40)	321 (78; 69 to 86)	17 (4; 3 to 7)
GP, ED and hospital admission	2376 (15; 15 to 16)	1647 (69; 67 to 71)	1091 (4; 4 to 4)	289 (26; 24 to 29)	59 (5; 4 to 7)	743 (68; 65 to 71)	383 (35; 32 to 39)	319 (83; 74 to 92)	18 (5; 3 to 7)
Total	15 739	9484 (60; 59 to 61)	28 970	13 677 (47; 47 to 48)	10 768 (37; 37 to 38)	4525 (16; 15 to 16)	7769 (27; 26 to 27)	5385 (69; 67 to 71)	253 (3; 3 to 4)

*Mutually exclusive groups.

†Total number of individuals presenting to service(s) over the study period

‡Event defined as a self-harm presentation across one or more services on a given date

§Percentage of presenting individuals

¶Presence of outpatient appointmnet within the susequent 30 days.

**Percentage of outpatient appointments under a mental health specialty

††Percentage of outpatient appointments under a paediatric specialty

ED, emergency department; GP, general practice.

There were 8665 admissions to hospital (singly or in combination with other settings; online supplementary table 5). Paediatric admission specialties made up the largest proportion of admissions (‘paediatric’ 39% (95% CI 38 to 40; n=3372); ‘general medicine’ 33% (95% CI 32 to 33; n=2813); ‘Psychiatric’ 1% (95% CI 1 to 1; n=69), of which 12% (95% CI 6 to 21; n=8) were under ‘child and adolescent psychiatry’; ‘surgical specialties’ 4% (95% CI 3 to 4; n=314); ‘ED surgical specialty’ 13% (95% CI 13 to 14; n=116); ‘other medical specialties’ 11% (95% CI 10 to 11; n=931)). Almost all individuals aged 10–14 years old were admitted under ‘paediatrics’ (99% (95% CI 98 to 99; n=2740)). Females were more commonly admitted under ‘paediatrics’ (females 47% (95% CI 46 to 49; n=2870); males 19% (95% CI 18 to 21; n=502)). A higher proportion of males were admitted under ‘general medicine’ (males 41% (95% CI 40 to 43; n=1077); females 29% (95% CI 27 to 30; n=1736)).

#### ED attendances and associated hospital admissions

**Figure 3:**
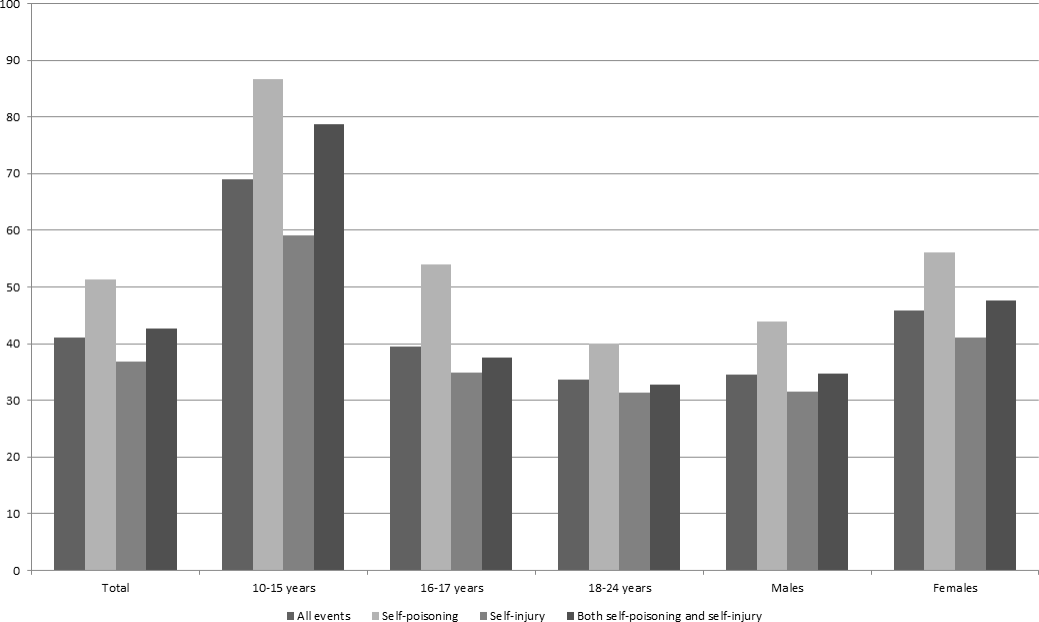
Percentage of emergency department attendances with an associated hospital admission.

Less than half of ED attendances were associated with a hospital admission. Age groups here are discussed in relation to the NICE guidance, split into 10–15, 16–18 and 19–24 years. Attendances in individuals aged 10–15 years old were more likely to be associated with an admission (10–15 years 69% (95% CI 67 to 71); 16–18 years 36% (95% CI 34 to 38); 19–24 years 34% (95% CI 33 to 36)) (figure 3 and online supplementary table 6).

Females are more likely than males to be admitted (46% (95% CI 45 to 47) vs 35% (95% CI 33 to 36)). This is most evident in those aged 10–15 years, where 76% (95% CI 74 to 78; n=1182) of females were admitted compared with 49% (95% CI 45 to 53) of males. This difference between sexes is less apparent in older age groups (16–18 years: females 40% (95% CI 38 to 42; n=2125) vs males 29% (95% CI 27 to 31; n=382); 19–24 years: females 34% (95% CI 33 to 36; n=1065) vs males 34% (95% CI 33 to 36; n=1095)).

Attendances for self-poisoning were more likely to be admitted than self-injuries (51% (95% CI 49 to 53) vs 37% (95% CI 36 to 38)). The highest proportion of admissions was seen in girls aged 10–15 years old attending for self-poisoning (90% (95% CI 87 to 93) admitted) compared with 69% (95% CI 59 to 78) of boys attending for self-poisoning of the same age.

#### Outpatient appointments

A third of SH events were associated with an outpatient appointment (table 2). The largest proportions of appointments were for those both ‘attending ED and admitted to hospital’. Only a quarter of those ‘attending ED only’ had an outpatient appointment within 30 days. The smallest proportion of associated outpatient appointments was seen in those presenting to ‘GP only’. The majority of outpatient appointments were under a mental health specialty. Only 3% (95% CI 3 to 4; n=253) were under a paediatric specialty.

#### ED attendances and associated outpatient appointments

**Figure 4:**
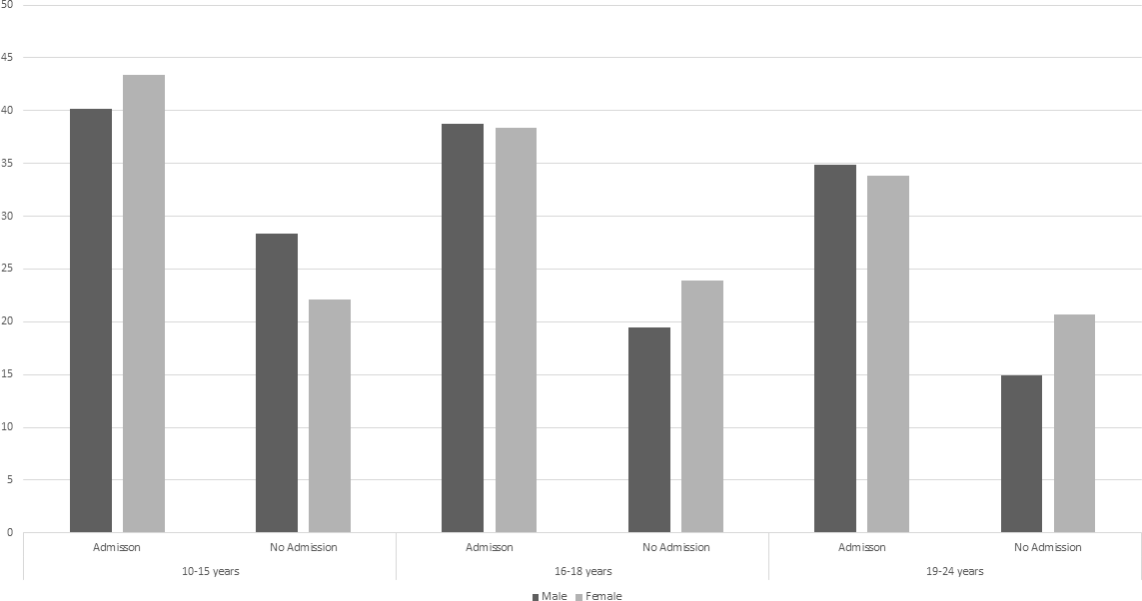
Percentage of emergency department attendances with an outpatient appointment in the subsequent 30 days by presence or absence of an associated hospital admission.

Only 27% (95% CI 26 to 28; n=3223) of ED attendances were associated with an outpatient appointment. Attendances associated with admission were more likely to have a subsequent outpatient appointment than those without (figure 4). In those aged 10–15 years, males are more likely than females to have an outpatient appointment without a hospital admission (males 28% (95% CI 23 to 34; n=81); females 22% (95% CI 18 to 27; n=81)). This is not seen in older age groups.

## Discussion

### Main findings

This is the first study of its kind to link SH across primary care, EDs and hospital admissions at the person level in the UK. Incidence of recorded SH is highest in primary care as found elsewhere.^9^ Previous research combining GP and hospital admissions found a significant increase in incidence in females aged 13–16 not reflected in older adolescents or males.^11^ This study supports the increase in females aged 10–14 across all settings. While incidence in GP has remained stable for males of this age, the breakdown by service and addition of ED data show ED attendances have more than doubled and hospital admissions have also significantly increased. While incidence in GP for those aged 15–19 remains stable, there is a significant increase in both ED attendances and admissions for females and in ED attendances for males. The absence of a corresponding increase in admissions for males of this age is a cause for concern. Raised awareness and disclosure or increasing medically severe SH in CYP may be resulting in increased presentation to hospital-based settings. Psychiatric admission is most likely when highly lethal methods are used.^8^ The increases in hospital admissions are potentially reflecting improved management of SH based on guidance recommending admission for individuals under 16.^1^ This does not explain increases in ED attendance. Admission specialties were examined, with 99% of those aged 10–15 admitted under paediatric specialties. For older age groups admission under ‘general medicine’ was more common.

SH method was found to vary over time by setting and sex. Self-injury increased significantly for both sexes, with a larger increase in females in keeping with ED^8^ and hospital admissions research also finding an increase in violent self-injury (eg, hanging).^26^ The current study additionally examined method of SH presenting to GPs and found a decrease in self-poisoning in both sexes and a significant increase over time for self-injury particularly for females.

We examined the demographics of individuals presenting to each service individually or in combination. A quarter of individuals present to ‘GP only’. A further 22% present to ‘ED only’, meaning almost half of individuals did not have a hospital admission. Females outnumber males in every combination of settings, with the exception of ‘ED only’ where 58% of individuals are male, underscoring the concerning disparity in admissions by sex. Self-injury is more likely to be used by males and less likely to result in admission if not medically required, possibly based on misconceptions regarding suicide risk.^6 27^

Less than half of ED attendances were associated with a hospital admission. Admission of individuals aged 16 and 17 years old is based on clinical judgement rather than stipulated in guidance.^8^ The results here support previous research demonstrating that under 16s are more likely to be admitted following ED attendance for SH than older age groups,^8^ in keeping with clinical guidance for this age group. The ratio of females to males attending ED with SH has been previously found to decrease with age, with those aged 12–14 most likely to be admitted.^28^ The results here additionally demonstrate that females are more likely to be admitted following ED attendance than males. This is most evident in those aged 10–15 years, where 76% of females attending ED are admitted compared with just 49% of males. This difference persists even in the case of self-poisoning. Males are more likely to leave ED before ongoing care recommendations can be made or to refuse treatment, which may partly underlie the lower levels of admissions.^8^

Less than a third of ED attendances had a subsequent outpatient appointment, with those admitted more likely to be seen in outpatients. This may indicate a need for better recording of liaison or crisis team contacts or reflect poor follow-up of patients who present with SH.^29^ In those aged 10–15 years, females were more likely than males to have an outpatient appointment with an admission; however, males were more likely than females to have an outpatient appointment without an admission. The majority of outpatient appointments were under a mental health specialty.

### Strengths and limitations

This study provides a comprehensive picture on which to base targeted intervention, resources and service provision. Derived from a large representative population of CYP studied over 12 years, results are generalisable to the rest of the UK.

Up to 14 diagnoses can be recorded per hospital admission, as such SH may not be the primary reason for admission. In self-poisoning the reason for admission is likely to be for medical treatment or monitoring of poison levels. We cannot be certain a hospital admission was a direct result of an ED attendance. This was based on hospital admission within 7 days. It was not possible to examine severity. While admission to hospital may reflect increased severity, there are multiple factors influencing whether or not someone is admitted (eg, age, method). Method was grouped broadly into ‘self-poisoning’ and ‘self-injury’. It was not possible to subdivide self-injury (eg, into hanging, traffic) due to insufficient numbers. While we examined admission by specialty, it was not possible to examine whether an individual was admitted to a general medical or psychiatric hospital.

Data from EDs are not available prior to 2009 and have less detailed coding of SH compared with GP and admissions data. ED data are collected from every ED in Wales. Prior to 2012 there were coding quality issues from some providers. As such data from 2009 to 2012 should be interpreted with caution particularly with regard to trends over time. ED trends over time presented here are in keeping with other research using ED data.^8^

Routinely collected data have limitations for research purposes, and the quality and completeness of data vary across data sets. We have attempted to minimise the impact of this by only including GPs that meet standards for data quality and using validated code lists.^9 12^ SH not resulting in presentation to services or where SH is discussed but not recorded will not be captured here. This is a common feature of all studies using routine data. These data are a reflection of contacts with the healthcare system, not of rates of SH in the community. GP coding behaviour changes over time.^24 30^ It is unclear whether the increase in SH reflects a genuine increase or if this is partially attributable to improved recognition and coding behaviour by clinicians and increased help-seeking by individuals.^20^

### Implications

GPs are an important setting for intervention. Despite this GPs may underestimate the prevalence of SH in young people, and have stated they would welcome training in communication with CYP and practical information about SH.^31^ The higher proportion of males in those who attend ED only combined with the higher overall suicide rate in males makes this an important setting for intervention in this hard-to-reach group. Tailored follow-up services should be considered. Qualitative research is needed to explore whether this reflects a preference of setting or delayed help-seeking until crisis. Improving the help-seeking skills of young males may prevent delayed presentation to emergency settings.^32^

Rates of ED attendance and hospital admissions are increasing for those aged 10–19 years. Individuals in younger age groups are often brought rather than initiating attendance themselves. Increases may reflect greater awareness of parents/carers and improved help-seeking, highlighting the increasing demand on resources. Older adolescents are more likely to initiate help-seeking in their own right. The higher rate of primary care contact for SH in older adolescents may be partially attributable to an increase in help-seeking with age.^33^

CYP regularly contact non-psychiatric specialist services. It is important professionals in these services are supported in their ability to identify CYP at risk, provide appropriate support and determine when referral to specialist services is required/urgent. Further training for wider National Health Service (NHS) staff with specific targets set in policy guidance (eg, ref 34) could improve management of those presenting with SH.

## Conclusions

This is the first UK study to explore trends over time and characteristics of patients with SH by healthcare setting, and the first to incorporate ED data into such analysis. Patients who are admitted to hospital make up only a small proportion of individuals presenting to services with SH, with a large proportion of individuals presenting to GP or ED only. Incidence of SH over time varies by age group, sex and service. Understanding patterns of presentation will inform service planning and configuration for follow-up care and could inform tailored support, for example for males in ED. Linked data provide important evidence to support the development of interventions across healthcare settings.
