# Supplementary material for: Self-harm presentation across healthcare settings by sex in young people: an e-cohort study using routinely collected linked healthcare data in Wales, UK
Source: Arch Dis Child. 2019 Oct 14;105(4):347–54. doi: 10.1136/archdischild-2019-317248 (PMC7146921; doi:10.1136/archdischild-2019-317248)
Supplement: Supplementary data [file archdischild-2019-317248supp001.pdf]

Appendix A

SAIL databank additional information

SAIL databank information governance and privacy procedures

The SAIL databank (www.saildatabank.com) is an expanding data repository (around 3 billion records) of privacy protected anonymised person-based linkable data from healthcare and public settings to support research. Robust policies, structures and controls are in place to protect privacy through reliable matching, anonymization and encryption process achieved in conjunction with the NHS Wales Information Service using a split file approach [13, 14]. This involves the separation of identifiable information from clinical content, identity matching and creation of anonymised linkage keys prior to reassembling and further encryption of datasets. This is described in further detail elsewhere [13, 14]. All data within the SAIL gateway are treated in accordance with the Data Protection Act (2017) and are compliant with the General Data Protection Regulation (GDPR).

Ethical approvals

Approval was granted from the Information Governance Review Panel (IGRP; approval number 0281). The IGRP oversees study approvals in line with permissions already granted to the analysis of data in the SAIL databank[13, 14]. SAIL databank Information Governance procedures specify that individual level data must be analysed in a data safe haven accessed through secure remote desktops. No person-level data may be removed from this safe haven. Aggregate data may be removed for publication following review to ensure that no personally identifiable data or small numbers (defined as n<5) are taken from this data safe haven.

Additional detail on datasets utilised in the current study

We used multiple patient level linked datasets:

1. The Welsh Demographic Service (WDS). A register of all individuals registered with a Welsh GP or who have ever had contact with the NHS
2. Welsh Index of Multiple Deprivation (WIMD). This dataset assigns all Lower Super Output Areas (LSOAs; geographic units comprised of around 1500 individuals) in Wales, a deprivation score derived from eight separate domains including income, employment and education (deprivation split into quintiles from 1 (most deprived) to

5 (least deprived) [15] These are calculated in a similar manner to the Index of Multiple Deprivation (IMD) used in England however domains are weighted differently in each region and as such the two indexes are not directly comparable. Deprivation indices are based on the LSOA of the individual patient based on their address data present in the WDS

3. General Practice Database (GPD). This dataset contains attendance and clinical information for all GP interactions including symptoms, diagnoses and prescriptions. GP practices opt-in to supplying SAIL. Currently 333 practices (out of 432 in Wales) contribute regularly updated data covering 77% of GP practices and 79% of the population (above 70% threshold for acceptable response in prevalence studies [16]). The population covered by the GPD are representative of the population as a whole in terms of gender (all Welsh practices 50% male; SAIL supplying practices 50% male) age range(all Welsh practices 20% aged 0-17 years, 60% aged 18-64 years, 20% aged 65+; SAIL supplying practices 20% aged 0-17 years, 61% aged 18-64 years, 19% aged 65) and deprivation indices (all Welsh practices 19% most deprived fifth, 18% least deprived fifth; SAIL supplying practices 20% most deprived fifth, 19% least deprived)
4. Emergency Department Dataset (EDDS). This dataset contains administrative and clinical information for all NHS Wales Accident and Emergency Department attendances (total of 34 sites including minor injuries units). Data is available from August 2009 onwards
5. Patient Episode Database for Wales (PEDW). This dataset contains attendance and clinical information for all NHS Wales hospital admissions (inpatient and day cases) including data regarding diagnoses, operations performed and admission specialty. A hospital admission in this study refers to period of continuous care whilst admitted to hospital (spell of care). This can encompass multiple episodes of consultant care and reflects a patient’s stay in hospital. This includes both inpatient and day cases and encompasses all consultant specialities. It is not possible to distinguish dedicated psychiatric hospitals from general hospitals however, data regarding the speciality under which an individual is admitted is available
6. Outpatient Data (OPD). This dataset contains attendance information for all NHS Wales hospital outpatient appointments from 2004 onwards

7. Office of National Statistics (ONS) deaths register. This is a register of all deaths relating to Welsh residents, including those that die outside of Wales and includes information regarding date and cause of death. Full details of these datasets can be found at www.saildatabank.com.
