# Supplementary material for: Self-harm presentation across healthcare settings by sex in young people: an e-cohort study using routinely collected linked healthcare data in Wales, UK
Source: Arch Dis Child. 2019 Oct 14;105(4):347–54. doi: 10.1136/archdischild-2019-317248 (PMC7146921; doi:10.1136/archdischild-2019-317248)
Supplement: Supplementary data [file archdischild-2019-317248supp002.pdf]

Appendix B: Read codes used to identify self-harm in GP data

Read Code	Description
14K1.	Intentional overdose of prescription only medication
SL...	Overdose of biological substance
SL90.	Antidepressant poisoning
SL900	Amitriptyline poisoning
SL901	Imipramine poisoning
SL902	Monoamine poisoning
SL903	Trazodone poisoning
SL90z	Anti-depressant poisoning NOS
TK...	Suicide and self inflicted injury
TK0..	Suicide + self inflicted poisoning by solid/liquid substances
TK00.	Suicide + self inflicted poisoning by analgesic/antipyretic
TK01.	Suicide + self inflicted poisoning by barbiturates
TK010	Suicide and self inflicted injury by Amylobarbitone
TK011	Suicide and self inflicted injury by Barbitone
TK014	Suicide and self inflicted injury by Phenobarbitone
TK02.	Suicide + self inflicted poisoning by oth sedatives/hypnotics
TK03.	Suicide + self inflicted poisoning tranquilliser/psychotropic
TK04.	Suicide + self inflicted poisoning by other drugs/medicines
TK05.	Suicide + self inflicted poisoning by drug or medicine NOS
TK06.	Suicide + self inflicted poisoning by agricultural chemical
TK07.	Suicide + self inflicted poisoning by corrosive/caustic subst

TK0z.	Suicide + self inflicted poisoning by solid/liquid subst NOS
TK1..	Suicide + self inflicted poisoning by gases in domestic use
TK10.	Suicide + self inflicted poisoning by gas via pipeline
TK11.	Suicide + self inflicted poisoning by liquified petrol gas
TK1y.	Suicide and self inflicted poisoning by other utility gas
TK1z.	Suicide + self inflicted poisoning by domestic gases NOS
TK2..	Suicide + self inflicted poisoning by other gases and vapours
TK20.	Suicide + self inflicted poisoning by motor veh exhaust gas
TK21.	Suicide and self inflicted poisoning by other carbon monoxide
TK2z.	Suicide + self inflicted poisoning by gases and vapours NOS
TK3..	Suicide + self inflicted injury by hang/strangulate/suffocate
TK30.	Suicide and self inflicted injury by hanging
TK31.	Suicide + self inflicted injury by suffocation by plastic bag
TK3y.	Suicide + self inflicted inj oth mean hang/strangle/suffocate
TK3z.	Suicide + self inflicted inj by hang/strangle/suffocate NOS
TK4..	Suicide and self inflicted injury by drowning
TK5..	Suicide and self inflicted injury by firearms and explosives
TK51.	Suicide and self inflicted injury by shotgun
TK52.	Suicide and self inflicted injury by hunting rifle
TK54.	Suicide and self inflicted injury by other firearm

TK5z.	Suicide and self inflicted injury by firearms/explosives NOS
TK6..	Suicide and self inflicted injury by cutting and stabbing
TK60.	Suicide and self inflicted injury by cutting
TK601	Self inflicted lacerations to wrist
TK61.	Suicide and self inflicted injury by stabbing
TK6z.	Suicide and self inflicted injury by cutting and stabbing NOS
TK7..	Suicide and self inflicted injury by jumping from high place
TK70.	Suicide+self inflicted injury-jump from residential premises
TK71.	Suicide+self inflicted injury-jump from oth manmade structure
TK72.	Suicide+self inflicted injury-jump from natural sites
TK7z.	Suicide+self inflicted injury-jump from high place NOS
TKx..	Suicide and self inflicted injury by other means
TKx0.	Suicide + self inflicted injury-jump/lie before moving object
TKx00	Suicide + self inflicted injury-jumping before moving object
TKx1.	Suicide and self inflicted injury by burns or fire
TKx2.	Suicide and self inflicted injury by scald
TKx3.	Suicide and self inflicted injury by extremes of cold
TKx4.	Suicide and self inflicted injury by electrocution
TKx5.	Suicide and self inflicted injury by crashing motor vehicle
TKx6.	Suicide and self inflicted injury by crashing of aircraft
TKx7.	Suicide and self inflicted injury caustic subst, excl poison

TKxy.	Suicide and self inflicted injury by other specified means
TKxz.	Suicide and self inflicted injury by other means NOS
TKy..	Late effects of self inflicted injury
TKz..	Suicide and self inflicted injury NOS
U2...	[X]Intentional self-harm
U20..	[X]Intentional self poisoning by and exposure to noxious substances
U200.	[X]Intentional self poisoning by and exposure to nonopioid analgesics
U2000	[X]Intentional self poisoning by and exposure to nonopioid analgesics, occurrence at home
U2001	[X]Intentional self poisoning by and exposure to nonopioid analgesics, occurrence in residential institution
U2002	[X]Intentional self poisoning by and exposure to nonopioid analgesics, occurrence at school, other institution and public administrative area
U2003	[X]Intentional self poisoning by and exposure to nonopioid analgesics, occurrence at sports and athletics area
U2004	[X]Intentional self poisoning by and exposure to nonopioid analgesics, occurrence on street and highway
U2005	[X]Intentional self poisoning by and exposure to nonopioid analgesics, occurrence at trade and service area
U2006	[X]Intentional self poisoning by and exposure to nonopioid analgesics, occurrence at industrial and construction area
U2007	[X]Intentional self poisoning by and exposure to nonopioid analgesics, occurrence on farm
U200y	[X]Intentional self poisoning by and exposure to nonopioid analgesics, occurrence at other specified place

U200z	[X]Intentional self poisoning by and exposure to nonopioid analgesics, occurrence at unspecified place
U201.	[X]Intentional self poisoning by and exposure to antiepileptics
U2010	[X]Intentional self poisoning by and exposure to antiepileptics, occurrence at home
U2011	[X]Intentional self poisoning by and exposure to antiepileptics, occurrence in residential institution
U2012	[X]Intentional self poisoning by and exposure to antiepileptics, occurrence at school, other institution and public administrative area
U2013	[X]Intentional self poisoning by and exposure to antiepileptics, occurrence at sports and athletics area
U2014	[X]Intentional self poisoning by and exposure to antiepileptics, occurrence on street and highway
U2015	[X]Intentional self poisoning by and exposure to antiepileptics, occurrence at trade and service area
U2016	[X]Intentional self poisoning by and exposure to antiepileptics, occurrence at industrial and construction area
U2017	[X]Intentional self poisoning by and exposure to antiepileptics, occurrence on farm
U201y	[X]Intentional self poisoning by and exposure to antiepileptics, occurrence at other specified place
U201z	[X]Intentional self poisoning by and exposure to antiepileptics, occurrence at unspecified place
U202.	[X]Intentional self poisoning by and exposure to sedative hypnotics
U2020	[X]Intentional self poisoning by and exposure to sedative hypnotics, occurrence at home
U2021	[X]Intentional self poisoning by and exposure to sedative hypnotics, occurrence in residential institution

U2022	[X]Intentional self poisoning by and exposure to sedative hypnotics, occurrence at school, other institution and public administrative area
U2023	[X]Intentional self poisoning by and exposure to sedative hypnotics, occurrence at sports and athletics area
U2024	[X]Intentional self poisoning by and exposure to sedative hypnotics, occurrence on street and highway
U2025	[X]Intentional self poisoning by and exposure to sedative hypnotics, occurrence at trade and service area
U2026	[X]Intentional self poisoning by and exposure to sedative hypnotics, occurrence at industrial and construction area
U2027	[X]Intentional self poisoning by and exposure to sedative hypnotics, occurrence on farm
U202y	[X]Intentional self poisoning by and exposure to sedative hypnotics, occurrence at other specified place
U202z	[X]Intentional self poisoning by and exposure to sedative hypnotics, occurrence at unspecified place
U203.	[X]Intentional self poisoning by and exposure to antiparkinson drugs
U2030	[X]Intentional self poisoning by and exposure to antiparkinson drugs, occurrence at home
U2031	[X]Intentional self poisoning by and exposure to antiparkinson drugs, occurrence in residential institution
U2032	[X]Intentional self poisoning by and exposure to antiparkinson drugs, occurrence at school, other institution and public administrative area
U2033	[X]Intentional self poisoning by and exposure to antiparkinson drugs, occurrence at sports and athletics area
U2034	[X]Intentional self poisoning by and exposure to antiparkinson drugs, occurrence on street and highway

U2035	[X]Intentional self poisoning by and exposure to antiparkinson drugs, occurrence at trade and service area
U2036	[X]Intentional self poisoning by and exposure to antiparkinson drugs, occurrence at industrial and construction area
U2037	[X]Intentional self poisoning by and exposure to antiparkinson drugs, occurrence on farm
U203y	[X]Intentional self poisoning by and exposure to antiparkinson drugs, occurrence at other specified place
U203z	[X]Intentional self poisoning by and exposure to antiparkinson drugs, occurrence at unspecified place
U204.	[X]Intentional self poisoning by and exposure to psychotropic drugs
U2040	[X]Intentional self poisoning by and exposure to psychotropic drugs, occurrence at home
U2041	[X]Intentional self poisoning by and exposure to psychotropic drugs, occurrence in residential institution
U2042	[X]Intentional self poisoning by and exposure to psychotropic drugs, occurrence at school, other institution and public administrative area
U2043	[X]Intentional self poisoning by and exposure to psychotropic drugs, occurrence at sports and athletics area
U2044	[X]Intentional self poisoning by and exposure to psychotropic drugs, occurrence on street and highway
U2045	[X]Intentional self poisoning by and exposure to psychotropic drugs, occurrence at trade and service area
U2046	[X]Intentional self poisoning by and exposure to psychotropic drugs, occurrence at industrial and construction area
U2047	[X]Intentional self poisoning by and exposure to psychotropic drugs, occurrence on farm

U204y	[X]Intentional self poisoning by and exposure to psychotropic drugs, occurrence at other specified place
U204z	[X]Intentional self poisoning by and exposure to psychotropic drugs, occurrence at unspecified place
U205.	[X]Intentional self poisoning by and exposure to narcotic drugs
U2050	[X]Intentional self poisoning by and exposure to narcotic drugs, occurrence at home
U2051	[X]Intentional self poisoning by and exposure to narcotic drugs, occurrence in residential institution
U2052	[X]Intentional self poisoning by and exposure to narcotic drugs, occurrence at school, other institution and public administrative area
U2053	[X]Intentional self poisoning by and exposure to narcotic drugs, occurrence at sports and athletics area
U2054	[X]Intentional self poisoning by and exposure to narcotic drugs, occurrence on street and highway
U2055	[X]Intentional self poisoning by and exposure to narcotic drugs, occurrence at trade and service area
U2056	[X]Intentional self poisoning by and exposure to narcotic drugs, occurrence at industrial and construction area
U2057	[X]Intentional self poisoning by and exposure to narcotic drugs, occurrence on farm
U205y	[X]Intentional self poisoning by and exposure to narcotic drugs, occurrence at other specified place
U205z	[X]Intentional self poisoning by and exposure to narcotic drugs, occurrence at unspecified place
U206.	[X]Intentional self poisoning by and exposure to hallucinogens
U2060	[X]Intentional self poisoning by and exposure to hallucinogens, occurrence at home

U2061	[X]Intentional self poisoning by and exposure to hallucinogens, occurrence in residential institution
U2062	[X]Intentional self poisoning by and exposure to hallucinogens, occurrence at school, other institution and public administrative area
U2063	[X]Intentional self poisoning by and exposure to hallucinogens, occurrence at sports and athletics area
U2064	[X]Intentional self poisoning by and exposure to hallucinogens, occurrence on street and highway
U2065	[X]Intentional self poisoning by and exposure to hallucinogens, occurrence at trade and service area
U2066	[X]Intentional self poisoning by and exposure to hallucinogens, occurrence at industrial and construction area
U2067	[X]Intentional self poisoning by and exposure to hallucinogens, occurrence on farm
U206y	[X]Intentional self poisoning by and exposure to hallucinogens, occurrence at other specified place
U206z	[X]Intentional self poisoning by and exposure to hallucinogens, occurrence at unspecified place
U207.	[X]Intentional self poisoning by and exposure to other autonomic drugs
U2070	[X]Intentional self poisoning by and exposure to other autonomic drugs, occurrence at home
U2071	[X]Intentional self poisoning by and exposure to other autonomic drugs, occurrence in residential institution
U2072	[X]Intentional self poisoning by and exposure to other autonomic drugs, occurrence at school, other institution and public administrative area
U2073	[X]Intentional self poisoning by and exposure to other autonomic drugs, occurrence at sports and athletics area

U2074	[X]Intentional self poisoning by and exposure to other autonomic drugs, occurrence on street and highway
U2075	[X]Intentional self poisoning by and exposure to other autonomic drugs, occurrence at trade and service area
U2076	[X]Intentional self poisoning by and exposure to other autonomic drugs, occurrence at industrial and construction area
U2077	[X]Intentional self poisoning by and exposure to other autonomic drugs, occurrence on farm
U207y	[X]Intentional self poisoning by and exposure to other autonomic drugs, occurrence at other specified place
U207z	[X]Intentional self poisoning by and exposure to other autonomic drugs, occurrence at unspecified place
U208.	[X]Intentional self poisoning by and exposure to other and unspecified drug, medicament and biological substance
U2080	[X]Intentional self poisoning by and exposure to other and unspecified drug, medicament and biological substance, occurrence at home
U2081	[X]Intentional self poisoning by and exposure to other and unspecified drug, medicament and biological substance, occurrence in residential institution
U2082	[X]Intentional self poisoning by and exposure to other and unspecified drug, medicament and biological substance, occurrence at school, other institution and public administrative area
U2083	[X]Intentional self poisoning by and exposure to other and unspecified drug, medicament and biological substance, occurrence at sports and athletics area
U2084	[X]Intentional self poisoning by and exposure to other and unspecified drug, medicament and biological substance, occurrence on street and highway
U2085	[X]Intentional self poisoning by and exposure to other and unspecified drug, medicament and

	biological substance, occurrence at trade and service area
U2086	[X]Intentional self poisoning by and exposure to other and unspecified drug, medicament and biological substance, occurrence at industrial and construction area
U2087	[X]Intentional self poisoning by and exposure to other and unspecified drug, medicament and biological substance, occurrence on farm
U208y	[X]Intentional self poisoning by and exposure to other and unspecified drug, medicament and biological substance, occurrence at other specified place
U208z	[X]Intentional self poisoning by and exposure to other and unspecified drug, medicament and biological substance, occurrence at unspecified place
U20A.	[X]Intentional self poisoning by and exposure to organic solvents and halogenated hydrocarbons and their vapours
U20A0	[X]Intentional self poisoning by and exposure to organic solvents and halogenated hydrocarbons and their vapours, occurrence at home
U20A1	[X]Intentional self poisoning by and exposure to organic solvents and halogenated hydrocarbons and their vapours, occurrence in residential institution
U20A2	[X]Intentional self poisoning by and exposure to organic solvents and halogenated hydrocarbons and their vapours, occurrence at school, other institution and public administrative area
U20A3	[X]Intentional self poisoning by and exposure to organic solvents and halogenated hydrocarbons and their vapours, halogens, occurrence at sports and athletics area
U20A4	[X]Intentional self poisoning by and exposure to organic solvents and halogenated hydrocarbons and their vapours, occurrence on street and highway
U20A5	[X]Intentional self poisoning by and exposure to organic solvents and halogenated hydrocarbons

	and their vapours, occurrence at trade and service area
U20A6	[X]Intentional self poisoning by and exposure to organic solvents and halogenated hydrocarbons and their vapours, occurrence at industrial and construction area
U20A7	[X]Intentional self poisoning by and exposure to organic solvents and halogenated hydrocarbons and their vapours, occurrence on farm
U20Ay	[X]Intentional self poisoning by and exposure to organic solvents and halogenated hydrocarbons and their vapours, occurrence at other specified place
U20Az	[X]Intentional self poisoning by and exposure to organic solvents and halogenated hydrocarbons and their vapours, halogens, occurrence at unspecified place
U20B.	[X]Intentional self poisoning by and exposure to other gas and vapours
U20B0	[X]Intentional self poisoning by and exposure to other gas and vapours, occurrence at home
U20B1	[X]Intentional self poisoning by and exposure to other gas and vapours, occurrence in residential institution
U20B2	[X]Intentional self poisoning by and exposure to other gas and vapour, occurrence at school, other institution and public administrative area
U20B3	[X]Intentional self poisoning by and exposure to other gas and vapour, occurrence at sports and athletics area
U20B4	[X]Intentional self poisoning by and exposure to other gas and vapour, occurrence on street and highway
U20B5	[X]Intentional self poisoning by and exposure to other gas and vapour, occurrence at trade and service area
U20B6	[X]Intentional self poisoning by and exposure to other gas and vapour, occurrence at industrial and construction area

U20B7	[X]Intentional self poisoning by and exposure to other gas and vapour, occurrence on farm
U20By	[X]Intentional self poisoning by and exposure to other gas and vapour, occurrence at other specified place
U20Bz	[X]Intentional self poisoning by and exposure to other gas and vapour, occurrence at unspecified place
U20C.	[X]Intentional self poisoning by and exposure to pesticides
U20C0	[X]Intentional self poisoning by and exposure to pesticides, occurrence at home
U20C1	[X]Intentional self poisoning by and exposure to pesticides, occurrence in residential institution
U20C2	[X]Intentional self poisoning by and exposure to pesticides, occurrence at school, other institution and public administrative area
U20C3	[X]Intentional self poisoning by and exposure to pesticides, occurrence at sports and athletics area
U20C4	[X]Intentional self poisoning by and exposure to pesticides, occurrence on street and highway
U20C5	[X]Intentional self poisoning by and exposure to pesticides, occurrence at trade and service area
U20C6	[X]Intentional self poisoning by and exposure to pesticides, occurrence at industrial and construction area
U20C7	[X]Intentional self poisoning by and exposure to pesticides, occurrence on farm
U20Cy	[X]Intentional self poisoning by and exposure to pesticides, occurrence at other specified place
U20Cz	[X]Intentional self poisoning by and exposure to pesticides, occurrence at unspecified place
U20y.	[X]Intentional self poisoning by and exposure to other and unspecified chemicals and noxious substances
U20y0	[X]Intentional self poisoning by and exposure to other and unspecified chemicals and noxious substances, occurrence at home

U20y1	[X]Intentional self poisoning by and exposure to other and unspecified chemicals and noxious substances, occurrence in residential institution
U20y2	[X]Intentional self poisoning by and exposure to other and unspecified chemicals and noxious substances, occurrence at school, other institution and public administrative area
U20y3	[X]Intentional self poisoning by and exposure to other and unspecified chemicals and noxious substances, occurrence at sports and athletics area
U20y4	[X]Intentional self poisoning by and exposure to other and unspecified chemicals and noxious substances, occurrence on street and highway
U20y5	[X]Intentional self poisoning by and exposure to other and unspecified chemicals and noxious substances, occurrence at trade and service area
U20y6	[X]Intentional self poisoning by and exposure to other and unspecified chemicals and noxious substances, occurrence at industrial and construction area
U20y7	[X]Intentional self poisoning by and exposure to other and unspecified chemicals and noxious substances, occurrence on farm
U20yy	[X]Intentional self poisoning by and exposure to other and unspecified chemicals and noxious substances, occurrence at other specified place
U20yz	[X]Intentional self poisoning by and exposure to other and unspecified chemicals and noxious substances, occurrence at unspecified place
U21..	[X]Intentional self harm by hanging, strangulation and suffocation
U210.	[X]Intentional self harm by hanging, strangulation and suffocation, occurrence at home
U211.	[X]Intentional self harm by hanging, strangulation and suffocation, occurrence in residential institution
U212.	[X]Intentional self harm by hanging, strangulation and suffocation, occurrence at

	school, other institution and public administrative area
U213.	[X]Intentional self harm by hanging, strangulation and suffocation, occurrence at sports and athletics area
U214.	[X]Intentional self harm by hanging, strangulation and suffocation, occurrence on street and highway
U215.	[X]Intentional self harm by hanging, strangulation and suffocation, occurrence at trade and service area
U216.	[X]Intentional self harm by hanging, strangulation and suffocation, occurrence at industrial and construction area
U217.	[X]Intentional self harm by hanging, strangulation and suffocation, occurrence on farm
U21y.	[X]Intentional self harm by hanging, strangulation and suffocation, occurrence at other specified place
U21z.	[X]Intentional self harm by hanging, strangulation and suffocation, occurrence at unspecified place
U22..	[X]Intentional self harm by drowning and submersion
U220.	[X]Intentional self harm by drowning and submersion, occurrence at home
U221.	[X]Intentional self harm by drowning and submersion, occurrence in residential institution
U222.	[X]Intentional self harm by drowning and submersion, occurrence at school, other institution and public administrative area
U223.	[X]Intentional self harm by drowning and submersion, occurrence at sports and athletics area
U224.	[X]Intentional self harm by drowning and submersion, occurrence on street and highway

U225.	[X]Intentional self harm by drowning and submersion, occurrence at trade and service area
U226.	[X]Intentional self harm by drowning and submersion, occurrence at industrial and construction area
U227.	[X]Intentional self harm by drowning and submersion, occurrence on farm
U22y.	[X]Intentional self harm by drowning and submersion, occurrence at other specified place
U22z.	[X]Intentional self harm by drowning and submersion, occurrence at unspecified place
U23..	[X]Intentional self harm by handgun discharge
U230.	[X]Intentional self harm by handgun discharge, occurrence at home
U231.	[X]Intentional self harm by handgun discharge, occurrence in residential institution
U232.	[X]Intentional self harm by handgun discharge, occurrence at school, other institution and public administrative area
U233.	[X]Intentional self harm by handgun discharge, occurrence at sports and athletics area
U234.	[X]Intentional self harm by handgun discharge, occurrence on street and highway
U235.	[X]Intentional self harm by handgun discharge, occurrence at trade and service area
U236.	[X]Intentional self harm by handgun discharge, occurrence at industrial and construction area
U237.	[X]Intentional self harm by handgun discharge, occurrence on farm
U23y.	[X]Intentional self harm by handgun discharge, occurrence at other specified place
U23z.	[X]Intentional self harm by handgun discharge, occurrence at unspecified place
U24..	[X]Intentional self harm by rifle, shotgun and larger firearm discharge
U240.	[X]Intentional self harm by rifle, shotgun and larger firearm discharge, occurrence at home

U241.	[X]Intentional self harm by rifle, shotgun and larger firearm discharge, occurrence in residential institution
U242.	[X]Intentional self harm by rifle, shotgun and larger firearm discharge, occurrence at school, other institution and public administrative area
U243.	[X]Intentional self harm by rifle, shotgun and larger firearm discharge, occurrence at sports and athletics area
U244.	[X]Intentional self harm by rifle, shotgun and larger firearm discharge, occurrence on street and highway
U245.	[X]Intentional self harm by rifle, shotgun and larger firearm discharge, occurrence at trade and service area
U246.	[X]Intentional self harm by rifle, shotgun and larger firearm discharge, occurrence at industrial and construction area
U247.	[X]Intentional self harm by rifle, shotgun and larger firearm discharge, occurrence on farm
U24y.	[X]Intentional self harm by rifle, shotgun and larger firearm discharge, occurrence at other specified place
U24z.	[X]Intentional self harm by rifle, shotgun and larger firearm discharge, occurrence at unspecified place
U25..	[X]Intentional self harm by other and unspecified firearm discharge
U250.	[X]Intentional self harm by other and unspecified firearm discharge, occurrence at home
U251.	[X]Intentional self harm by other and unspecified firearm discharge, occurrence in residential institution
U252.	[X]Intentional self harm by other and unspecified firearm discharge, occurrence at school, other institution and public administrative area

U253.	[X]Intentional self harm by other and unspecified firearm discharge, occurrence at sports and athletics area
U254.	[X]Intentional self harm by other and unspecified firearm discharge, occurrence on street and highway
U255.	[X]Intentional self harm by other and unspecified firearm discharge, occurrence at trade and service area
U256.	[X]Intentional self harm by other and unspecified firearm discharge, occurrence at industrial and construction area
U257.	[X]Intentional self harm by other and unspecified firearm discharge, occurrence on farm
U25y.	[X]Intentional self harm by other and unspecified firearm discharge, occurrence at other specified place
U25z.	[X]Intentional self harm by other and unspecified firearm discharge, occurrence at unspecified place
U26..	[X]Intentional self harm by explosive material
U260.	[X]Intentional self harm by explosive material, occurrence at home
U261.	[X]Intentional self harm by explosive material, occurrence in residential institution
U262.	[X]Intentional self harm by explosive material, occurrence at school, other institution and public administrative area
U263.	[X]Intentional self harm by explosive material, occurrence at sports and athletics area
U264.	[X]Intentional self harm by explosive material, occurrence on street and highway
U265.	[X]Intentional self harm by explosive material, occurrence at trade and service area
U266.	[X]Intentional self harm by explosive material, occurrence at industrial and construction area
U267.	[X]Intentional self harm by explosive material, occurrence on farm

U26y.	[X]Intentional self harm by explosive material, occurrence at other specified place
U26z.	[X]Intentional self harm by explosive material, occurrence at unspecified place
U27..	[X]Intentional self harm by smoke, fire and flames
U270.	[X]Intentional self harm by smoke, fire and flames, occurrence at home
U271.	[X]Intentional self harm by smoke, fire and flames, occurrence in residential institution
U272.	[X]Intentional self harm by smoke, fire and flames, occurrence at school, other institution and public administrative area
U273.	[X]Intentional self harm by smoke, fire and flames, occurrence at sports and athletics area
U274.	[X]Intentional self harm by smoke, fire and flames, occurrence on street and highway
U275.	[X]Intentional self harm by smoke, fire and flames, occurrence at trade and service area
U276.	[X]Intentional self harm by smoke, fire and flames, occurrence at industrial and construction area
U277.	[X]Intentional self harm by smoke, fire and flames, occurrence on farm
U27y.	[X]Intentional self harm by smoke, fire and flames, occurrence at other specified place
U27z.	[X]Intentional self harm by smoke, fire and flames, occurrence at unspecified place
U28..	[X]Intentional self harm by steam, hot vapours and hot objects
U280.	[X]Intentional self harm by steam, hot vapours and hot objects, occurrence at home
U281.	[X]Intentional self harm by steam, hot vapours and hot objects, occurrence in residential institution
U282.	[X]Intentional self harm by steam, hot vapours and hot objects, occurrence at school, other institution and public administrative area

U283.	[X]Intentional self harm by steam, hot vapours and hot objects, occurrence at sports and athletics area
U284.	[X]Intentional self harm by steam, hot vapours and hot objects, occurrence on street and highway
U285.	[X]Intentional self harm by steam, hot vapours and hot objects, occurrence at trade and service area
U286.	[X]Intentional self harm by steam, hot vapours and hot objects, occurrence at industrial and construction area
U287.	[X]Intentional self harm by steam, hot vapours and hot objects, occurrence on farm
U28y.	[X]Intentional self harm by steam, hot vapours and hot objects, occurrence at other specified place
U28z.	[X]Intentional self harm by steam, hot vapours and hot objects, occurrence at unspecified place
U29..	[X]Intentional self harm by sharp object
U290.	[X]Intentional self harm by sharp object, occurrence at home
U291.	[X]Intentional self harm by sharp object, occurrence in residential institution
U292.	[X]Intentional self harm by sharp object, occurrence at school, other institution and public administrative area
U293.	[X]Intentional self harm by sharp object, occurrence at sports and athletics area
U294.	[X]Intentional self harm by sharp object, occurrence on street and highway
U295.	[X]Intentional self harm by sharp object, occurrence at trade and service area
U296.	[X]Intentional self harm by sharp object, occurrence at industrial and construction area
U297.	[X]Intentional self harm by sharp object, occurrence on farm

U29y.	[X]Intentional self harm by sharp object, occurrence at other specified place
U29z.	[X]Intentional self harm by sharp object, occurrence at unspecified place
U2A..	[X]Intentional self harm by blunt object
U2A0.	[X]Intentional self harm by blunt object, occurrence at home
U2A1.	[X]Intentional self harm by blunt object, occurrence in residential institution
U2A2.	[X]Intentional self harm by blunt object, occurrence at school, other institution and public administrative area
U2A3.	[X]Intentional self harm by blunt object, occurrence at sports and athletics area
U2A4.	[X]Intentional self harm by blunt object, occurrence on street and highway
U2A5.	[X]Intentional self harm by blunt object, occurrence at trade and service area
U2A6.	[X]Intentional self harm by blunt object, occurrence at industrial and construction area
U2A7.	[X]Intentional self harm by blunt object, occurrence on farm
U2Ay.	[X]Intentional self harm by blunt object, occurrence at other specified place
U2Az.	[X]Intentional self harm by blunt object, occurrence at unspecified place
U2B..	[X]Intentional self harm by jumping from a high place
U2B0.	[X]Intentional self harm by jumping from a high place, occurrence at home
U2B1.	[X]Intentional self harm by jumping from a high place, occurrence in residential institution
U2B2.	[X]Intentional self harm by jumping from a high place, occurrence at school, other institution and public administrative area
U2B3.	[X]Intentional self harm by jumping from a high place, occurrence at sports and athletics area

U2B4.	[X]Intentional self harm by jumping from a high place, occurrence on street and highway
U2B5.	[X]Intentional self harm by jumping from a high place, occurrence at trade and service area
U2B6.	[X]Intentional self harm by jumping from a high place, occurrence at industrial and construction area
U2B7.	[X]Intentional self harm by jumping from a high place, occurrence on farm
U2By.	[X]Intentional self harm by jumping from a high place, occurrence at other specified place
U2Bz.	[X]Intentional self harm by jumping from a high place, occurrence at unspecified place
U2C..	[X]Intentional self harm by jumping or lying before moving object
U2C0.	[X]Intentional self harm by jumping or lying before moving object, occurrence at home
U2C1.	[X]Intentional self harm by jumping or lying before moving object, occurrence in residential institution
U2C2.	[X]Intentional self harm by jumping or lying before moving object, occurrence at school, other institution and public administrative area
U2C3.	[X]Intentional self harm by jumping or lying before moving object, occurrence at sports and athletics area
U2C4.	[X]Intentional self harm by jumping or lying before moving object, occurrence on street and highway
U2C5.	[X]Intentional self harm by jumping or lying before moving object, occurrence at trade and service area
U2C6.	[X]Intentional self harm by jumping or lying before moving object, occurrence at industrial and construction area
U2C7.	[X]Intentional self harm by jumping or lying before moving object, occurrence on farm

U2Cy.	[X]Intentional self harm by jumping or lying before moving object, occurrence at other specified place
U2Cz.	[X]Intentional self harm by jumping or lying before moving object, occurrence at unspecified place
U2D..	[X]Intentional self harm by crashing of motor vehicle
U2D0.	[X]Intentional self harm by crashing of motor vehicle, occurrence at home
U2D1.	[X]Intentional self harm by crashing of motor vehicle, occurrence in residential institution
U2D2.	[X]Intentional self harm by crashing of motor vehicle, occurrence at school, other institution and public administrative area
U2D3.	[X]Intentional self harm by crashing of motor vehicle, occurrence at sports and athletics area
U2D4.	[X]Intentional self harm by crashing of motor vehicle, occurrence on street and highway
U2D5.	[X]Intentional self harm by crashing of motor vehicle, occurrence at trade and service area
U2D6.	[X]Intentional self harm by crashing of motor vehicle, occurrence at industrial and construction area
U2D7.	[X]Intentional self harm by crashing of motor vehicle, occurrence on farm
U2Dy.	[X]Intentional self harm by crashing of motor vehicle, occurrence at other specified place
U2Dz.	[X]Intentional self harm by crashing of motor vehicle, occurrence at unspecified place
U2E..	[X]Self mutilation
U2y..	[X]Intentional self harm by other specified means
U2y0.	[X]Intentional self harm by other specified means, occurrence at home
U2y1.	[X]Intentional self harm by other specified means, occurrence in residential institution

U2y2.	[X]Intentional self harm by other specified means, occurrence at school, other institution and public administrative area
U2y3.	[X]Intentional self harm by other specified means, occurrence at sports and athletics area
U2y4.	[X]Intentional self harm by other specified means, occurrence on street and highway
U2y5.	[X]Intentional self harm by other specified means, occurrence at trade and service area
U2y6.	[X]Intentional self harm by other specified means, occurrence at industrial and construction area
U2y7.	[X]Intentional self harm by other specified means, occurrence on farm
U2yy.	[X]Intentional self harm by other specified means, occurrence at other specified place
U2yz.	[X]Intentional self harm by other specified means, occurrence at unspecified place
U2z..	[X]Intentional self harm by unspecified means
U2z0.	[X]Intentional self harm by unspecified means, occurrence at home
U2z1.	[X]Intentional self harm by unspecified means, occurrence in residential institution
U2z2.	[X]Intentional self harm by unspecified means, occurrence at school, other institution and public administrative area
U2z3.	[X]Intentional self harm by unspecified means, occurrence at sports and athletics area
U2z4.	[X]Intentional self harm by unspecified means, occurrence on street and highway
U2z5.	[X]Intentional self harm by unspecified means, occurrence at trade and service area
U2z6.	[X]Intentional self harm by unspecified means, occurrence at industrial and construction area
U2z7.	[X]Intentional self harm by unspecified means, occurrence on farm
U2zy.	[X]Intentional self harm by unspecified means, occurrence at other specified place

U2zz.	[X]Intentional self harm by unspecified means, occurrence at unspecified place
U41..	[X]Hanging strangulation + suffocation undetermined intent
U44..	[X]Rifle shotgun+larger firearm discharge undetermin intent
U45..	[X]Other+unspecified firearm discharge undetermined intent
U4B..	[X]Falling jumping/pushed from high place undeterm intent
U4Bz.	[X]Fall jump/push frm high plce undt intnt occ unspecif plce
U72..	[X]Sequel intentn self-harm assault+event of undeterm intent
U720.	[X]Sequelae of intentional self-harm
ZRLfC12	Health of the Nation Outcome Scales item 2 – nonaccidental self-injury
ZX...	Self-harm
ZX1..	Self-injurious behaviour
ZX11.	Biting self
ZX12.	Burning self
ZX13.	Cutting self
ZX15.	Drowning self
ZX18.	Hanging self
ZX19.	Hitting self
ZX191	Punching self
ZX192	Slapping self
ZX1B.	Jumping from height
ZX1B1	Jumping from building
ZX1B2	Jumping from bridge
ZX1B3	Jumping from cliff
ZX1C.	Nipping self
ZX1E.	Pinching self

ZX1G.	Scratches self
ZX1H.	Self-asphyxiation
ZX1H1	Self-strangulation
ZX1H2	Self-suffocation
ZX1I.	Self-scalding
ZX1J.	Self-electrocution
ZX1K.	Self-incineration
ZX1L.	Self-mutilation
ZX1L1	Self-mutilation of hands
ZX1L2	Self-mutilation of genitalia
ZX1L3	Self-mutilation of penis
ZX1L6	Self-mutilation of ears
ZX1LD	[X]Self mutilation
ZX1M.	Shooting self
ZX1N.	Stabbing self
ZX1Q.	Throwing self in front of train
ZX1R.	Throwing self in front of vehicle
ZX1S.	Throwing self onto floor
