# Supplementary material for: Self-harm presentation across healthcare settings by sex in young people: an e-cohort study using routinely collected linked healthcare data in Wales, UK
Source: Arch Dis Child. 2019 Oct 14;105(4):347–54. doi: 10.1136/archdischild-2019-317248 (PMC7146921; doi:10.1136/archdischild-2019-317248)
Supplement: Supplementary data [file archdischild-2019-317248supp003.pdf]

Appendix C: Identification of appropriate codes in ED data

Emergency Department

All attendances recorded under the attendance group 'deliberate self-harm' were included unless the sole diagnosis was overdose of alcohol.

Due to the nature of the coding system employed in the ED dataset it is not possible to simply choose codes equivalent to those available in READ or ICD 10. Additional analysis of events recorded under attendance group 'undetermined intent' was conducted in order to identify diagnoses related to self-harm.

All ED attendance from 2011-2015 were identified (n = 59344). An event was defined as a date in which an individual attended ED and was recorded as attendance group 'undetermined intent'. If multiple attendances in one day were recorded this would still only be counted as one event.

Admission to hospital within seven days of ED attendance was employed as a measure of whether an ED attendance resulted in admission to hospital. A period of seven days was chosen to allow for a possible 48 hours spent in ED and for any delays in data recording. Of the ED events identified 8251 were followed by a hospital admission within seven days. Of these admissions 666 had a diagnosis of self0harm recorded within that spell of care.

The ED diagnoses for the 666 cases that were subsequently admitted to hospital with self-harm were identified. An ED diagnosis of overdose (excluding alcohol) was recorded in 71% (n=470) of cases. Lacerations to wrists and forearms were recorded in 5% (n=35) of events.

Including both those with diagnoses of overdose (excluding alcohol) and laceration to the wrist or forearm accounts for 75% (n=505) of ED events recorded as undetermined intent where there is an admission to hospital within 7 days.

No other single EDDS diagnosis makes up a sufficiently large group (next largest is psychiatric disorder unspecified n = 17) so have only included overdose or laceration to wrist and forearm in the undetermined intent group.

Self-harm recorded if attendance group = 'deliberate self-harm' or if attendance group = undetermined intent alongside the following diagnosis codes

Poisoning or overdose
10B Prescribed Drug
10C Non-prescribed/purchased drug
10D Illicit Drug
10Z Poisoning or Overdose,
Unspecified
Wound
01A Laceration, with corresponding anatomical areas
205 Forearm
206 Wrist
