# Supplementary figures and images for: Self-harm presentation across healthcare settings by sex in young people: an e-cohort study using routinely collected linked healthcare data in Wales, UK
Source: Arch Dis Child. 2019 Oct 14;105(4):347–54. doi: 10.1136/archdischild-2019-317248 (PMC7146921; doi:10.1136/archdischild-2019-317248)

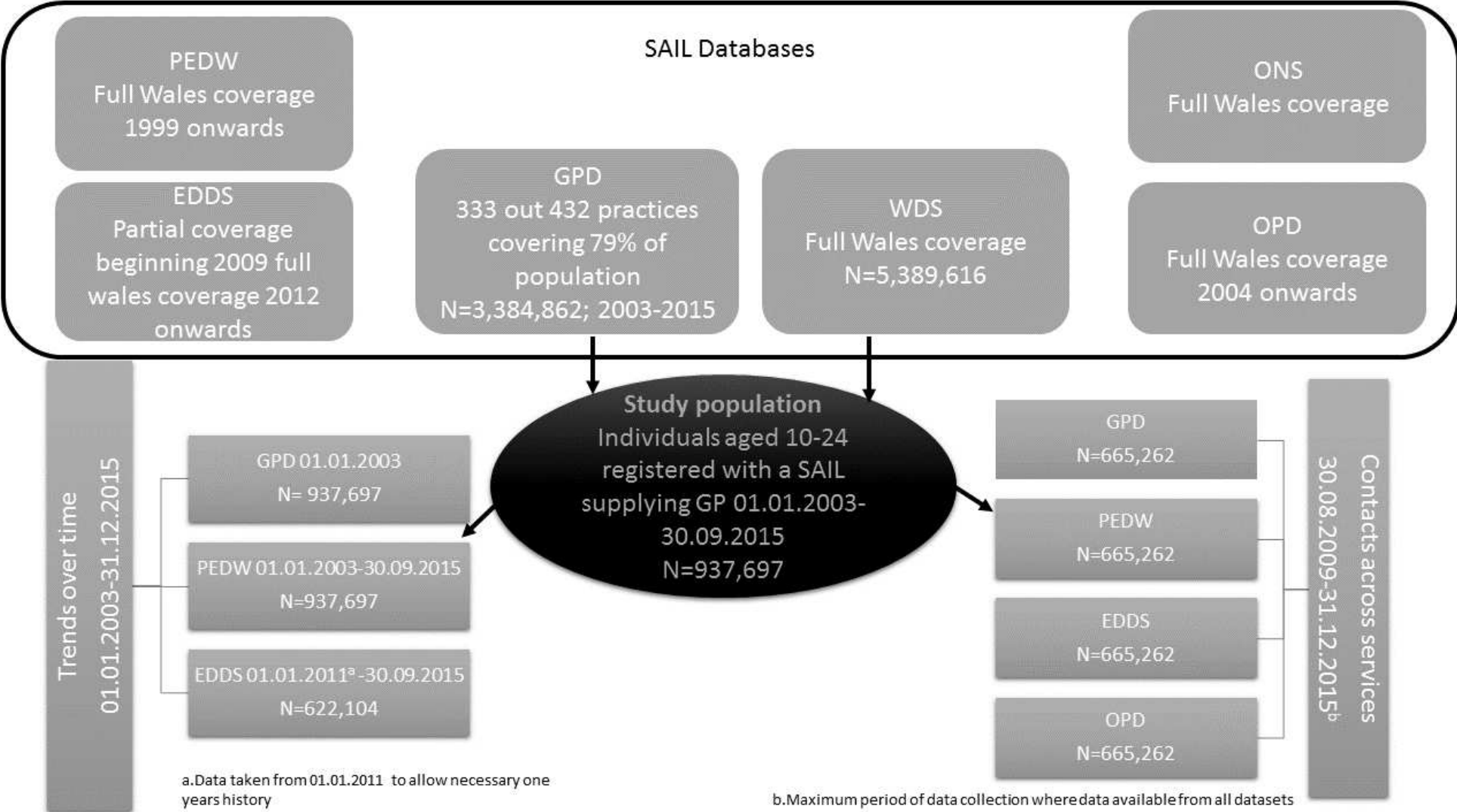

Supplement: Supplementary data [file archdischild-2019-317248supp004.pdf]
