# Supplementary material for: Self-harm presentation across healthcare settings by sex in young people: an e-cohort study using routinely collected linked healthcare data in Wales, UK
Source: Arch Dis Child. 2019 Oct 14;105(4):347–54. doi: 10.1136/archdischild-2019-317248 (PMC7146921; doi:10.1136/archdischild-2019-317248)
Supplement: Supplementary data [file archdischild-2019-317248supp005.pdf]

Appendix D

Categorisation of Admission Specialties

Surgical Specialties

100 General Surgery

101 Urology

102 Transplantation Surgery

103 Breast Surgery

104 Colorectal Surgery

105 Hepatobiliary & Pancreatic Surgery

106 Upper Gastrointestinal Surgery

107 Vascular Surgery

108 Spinal Surgery Service

110 Trauma & Orthopaedics

120 ENT

130 Ophthalmology

140 Oral Surgery

141 Restorative Dentistry

142 Paediatric Dentistry

143 Orthodontics

144 Maxillo-Facial Surgery

150 Neurosurgery

160 Plastic Surgery

161 Burns Care

170 Cardiothoracic Surgery

172 Cardiac Surgery

173 Thoracic Surgery

174 Cardiothoracic Transplantation

191 Pain Management

199 Non-UK provider; specialty function not know, treatment mainly surgical (only applicable for overseas providers)

Accident and Emergency Surgical Specialty

180 Accident & Emergency

Paediatrics

211 Paediatric Urology

212 Paediatric Transplantation Surgery

213 Paediatric Gastrointestinal Surgery

214 Paediatric Trauma And Orthopaedics

215 Paediatric Ear Nose And Throat

216 Paediatric Ophthalmology

217 Paediatric Maxillo-Facial Surgery

218 Paediatric Neurosurgery

219 Paediatric Plastic Surgery

220 Paediatric Burns Care

221 Paediatric Cardiac Surgery

222 Paediatric Thoracic Surgery

223 Paediatric Epilepsy

241 Paediatric Pain Management

242 Paediatric Intensive Care

251 Paediatric Gastroenterology

252 Paediatric Endocrinology

253 Paediatric Clinical Haematology

254 Paediatric Audiological Medicine

255 Paediatric Clinical Immunology And Allergy Service

256 Paediatric Infectious Diseases

257 Paediatric Dermatology

258 Paediatric Respiratory Medicine

259 Paediatric Nephrology

260 Paediatric Medical Oncology

261 Paediatric Metabolic Disease

262 Paediatric Rheumatology

263 Paediatric Diabetic Medicine

264 Paediatric Cystic Fibrosis

280 Paediatric Interventional Radiology

290 Community Paediatrics

291 Paediatric Neuro-Disability

171 Paediatric Surgery

321 Paediatric Cardiology

420 Paediatrics

421 Paediatric Neurology

Psychiatry

700 Learning Disability

710 Adult Mental Illness

711 Child & Adolescent Psychiatry

712 Forensic Psychiatry

713 Psychotherapy

715 Old Age Psychiatry

720 Eating Disorders

721 Addiction Services

722 Liaison Psychiatry

723 Psychiatric Intensive Care

724 Perinatal Psychiatry

725 Mental Health Recovery And Rehabilitation Service

726 Mental Health Dual Diagnosis Service

727 Dementia Assessment Service

656 Clinical Psychology

General Medicine

300 General Medicine

Other Medical Specialities

190 Anaesthetics

192 Critical Care Medicine

301 Gastroenterology

302 Endocrinology

303 Clinical Haematology

304	Clinical Physiology
305	Clinical Pharmacology
306	Hepatology
307	Diabetic Medicine
308	Blood And Marrow Transplantation
309	Haemophilia Service
310	Audiological Medicine
311	Clinical Genetics
312	Clinical Cytogenetics and Molecular Genetics
313	Clinical Immunology and Allergy
314	Rehabilitation Service
315	Palliative Medicine
316	Clinical Immunology
317	Allergy Service
318	Intermediate Care
319	Respite Care
320	Cardiology
322	Clinical Microbiology
323	Spinal Injuries
324	Anticoagulant Service
325	Sport And Exercise Medicine
327	Cardiac Rehabilitation
328	Stroke Medicine
329	Transient Ischaemic Attack
330	Dermatology
331	Congenital Heart Disease Service
340	Respiratory Medicine
341	Respiratory Physiology
342	Programmed Pulmonary Rehabilitation
343	Adult Cystic Fibrosis Service
344	Complex Specialised Rehabilitation Service

345 Specialist Rehabilitation Service

346 Local Specialist Rehabilitation Service

350 Infectious Diseases

352 Tropical Medicine

360 Genitourinary Medicine

361 Nephrology

370 Medical Oncology

371 Nuclear Medicine

400 Neurology

401 Clinical Neurophysiology

410 Rheumatology

422 Neonatology

424 Well Babies

430 Geriatric Medicine

450 Dental Medicine Specialties

460 Medical Ophthalmology

499 Non-UK provider; specialty function not known, treatment mainly medical (only applicable for overseas providers)

501 Obstetrics

502 Gynaecology

503 Gynaecological Oncology

510 Obstetrics – AN (outpatients)

520 Obstetrics - PN (outpatients)

560 Midwifery Service

610 GP Maternity

620 GP Other

650 Physiotherapy

651 Occupational Therapy

652 Speech And Language Therapy

653 Podiatry

654 Dietetics

655	Orthoptics
657	Prosthetics
658	Orthotics
659	Drama Therapy
660	Art Therapy
661	Music Therapy
662	Optometry
663	Podiatric Surgery
800	Clinical Oncology (previously Radiotherapy)
810	Radiology
811	Interventional Radiology
812	Diagnostic Imaging
820	General Pathology
821	Blood Transfusion
822	Chemical Pathology
823	Haematology (non-clinical)
824	Histopathology
830	Immunopathology
831	Medical Microbiology
832	Neuropathology
834	Medical Virology
840	Audiology
900	Community Medicine
901	Occupational Medicine
920	Diabetic Education Service
950	Nursing
990	Joint Consultant Clinics
998	Diagnostic*
999	Allied Health Professional (AHP) Services*
