# Supplementary material for: Self-harm presentation across healthcare settings by sex in young people: an e-cohort study using routinely collected linked healthcare data in Wales, UK
Source: Arch Dis Child. 2019 Oct 14;105(4):347–54. doi: 10.1136/archdischild-2019-317248 (PMC7146921; doi:10.1136/archdischild-2019-317248)
Supplement: Supplementary data [file archdischild-2019-317248supp006.pdf]

Supplementary Tables

Supplementary Table 1 Cohort Demographics

	%(95% CI; n)	Average age at onset of follow-up(SD)	Average person years follow-up(SD)
Males	50.4(50.3-50.6; n=472923)	14(4.9)	6.0(3.8)
Females	49.7(49.5-49.8; n=465774)	15(4.9)	5.9(3.8)
**All**		**15(4.9)**	**5.9(.8)**

		**%(95CI; n)**
Deprivation	(least deprived)	20.4(20.3-20.5; 191578)
	4	17.8(17.8-17.9; 167291)
	3	17.6(17.5-17.7; 164850)
	2	16.9(16.8-17; 158470)
	(Most deprived)	20.5(20.4-20.6; 192448)
	Unknown	6.7(6.7-6.8; 63060)

Supplementary Table 2 n; incidence per 1000 PYAR(95% CI) and IRRs (95%CI) of incident presentations for self-harm by age group and service in females

	10-14 years						15-19 years						20-24 years					
	GP		ED attendances		Hospital admissions		GP		ED attendances		Hospital admissions		GP		ED attendances		Hospital admissions	
	n; inc[a]	IRR	n; inc[a]	IRR	n; inc[a]	IRR	n; inc[a]	IRR	n; inc[a]	IRR	n; inc[a]	IRR	n; inc[a]	IRR	n; inc[a]	IRR	n	IRR
2003	285; 4(3.6-4.5)	Ref[b]			125; 1.8(1.5-2.1)	Ref[b]	562; 8.9(8.2-9.7)				272; 4.3(3.8-4.9)	Ref[b]	288; 5.3(4.7-5.9)	Ref[b]			153; 2.8(2.4-3.3)	Ref[b]
2004	277; 3.7(3.3-4.2)	0.9(0.8-1.1)			134; 1.8(1.5-2.1)	1.0(0.9-1.2)	635; 9.2(8.5-10.0)	1.0(1.0-1.1)			298; 4.3(3.9-4.9)	1.0(0.9-1.1)	349; 5.5(4.9-6.1)	1.0(0.9-1.2)			156; 2.5(2.1-2.9)	0.8(0.9-1.0)
2005	294; 3.9(3.5-4.4)	1.0(0.8-1.3)			182; 2.4(2.1-2.8)	1.4(1.1-1.7)	648; 9.2(8.5-9.9)	1.0(1.0-1.1)			353; 5(4.5-5.5)	1.2(1.0-1.3)	376; 5.7(5.1-6.3)	1.1(1.0-1.2)			184; 2.8(2.4-3.2)	1.0(0.8-1.3)
2006	292; 3.9(3.5-4.4)	1.0(0.8-1.1)			202; 2.7(2.4-3.1)	1.6(1.3-1.8)	645; 8.9(8.3-9.7)	1.0(0.9-1.1)			361; 5(4.5-5.5)	1.2(1.0-1.3)	374; 5.6(5.0-6.1)	1.0(0.9-1.2)			215; 3.2(2.8-3.6)	1.1(1.0-1.3)
2007	275; 3.7(3.3-4.2)	0.9(0.8-1.1)			189; 2.6(2.2-3.0)	1.5(1.3-1.6)	752; 10.3(9.6-11.1)	1.2(1.1-1.2)			433; 5.9(5.4-6.5)	1.4(1.2-1.5)	436; 6.4(5.8-7.0)	1.2(1.1-1.3)			221; 3.2(2.8-3.7)	1.2(1.0-1.4)
2008	273; 3.8(3.3-4.2)	0.9(0.8-1.1)			159; 2.2(1.9-2.6)	1.3(1.1-1.5)	731; 10(9.3-10.8)	1.1(1.0-1.2)			401; 5.5(5.0-6.1)	1.3(1.1-1.5)	454; 6.6(6.0-7.2)	1.2(1.1-1.4)			246; 3.6(3.1-4.0)	1.3(1.1-1.4)
2009	246; 3.4(3.0-3.9)	0.9(0.7-1.0)			169; 2.4(2.0-2.7)	1.3(1.2-1.5)	605; 8.4(7.7-9.1)	1.0(0.9-1.0)			333; 4.6(4.1-5.2)	1.1(1.0-1.2)	397; 5.8(5.2-6.3)	1.1(1.0-1.2)			187; 2.7(2.3-3.1)	1.0(0.81-1.2)
2010	246; 3.5(3.1-4.0)	0.9(0.7-1.0)			156; 2.2(1.9-2.6)	1.3(1.1-1.5)	622; 8.8(8.2-9.6)	1.0(0.9-1.0)			313; 4.4(4-5)	1.0(0.9-1.1)	389; 5.7(5.1-6.3)	1.1(1.0-1.2)			189; 2.8(2.4-3.2)	1.0(0.8-1.3)
2011	243; 3.5(3.1-4.0)	0.9(0.8-1.0)	133; 1.9(1.6-2.3)	Ref[b]	158; 2.3(1.9-2.7)	1.3(1.1-1.5)	601; 8.8(8.1-9.5)	1.0(0.9-1.1)	411; 6(5.4-6.6)	Ref[b]	339; 4.9(4.4-5.5)	1.1(1.0-1.3)	361; 5.3(4.8-5.9)	1.0(0.9-1.1)	243; 3.6(3.1-4.1)	Ref[b]	204; 3.0(2.6-3.4)	1.0(0.9-1.2)
2012	337; 5.0(4.5-	1.2(1.0-1.5)	160; 2.4(2.0-	1.3(0.9-1.7)	224; 3.3(2.9-	1.9(1.6-2.3)	559; 8.3(7.6-	0.9(0.8-1.0)	410; 6.1(5.5-	1.0(0.9-1.1)	301; 4.5(4.0-	1.0(0.9-1.2)	372; 5.5(5.0-	1.0(1.0-1.1)	283; 4.2(3.7-	1.2(1.0-1.3)	178; 2.6(2.3-	0.9(0.8-1.1)

	n; inc[a]	IRR	n; inc[a]	IRR	n; inc[a]	IRR	n; inc[a]	IRR	n; inc[a]	IRR	n; inc[a]	IRR	n; inc[a]	IRR	n; inc[a]	IRR	n	IRR
	5.5)		2.8)		3.8)		9.0)		6.7)		5.0)		6.1)		4.7)		3.1)	
2013	424; 6.4(5.8-7.1)	1.6(1.4-1.9)	192; 2.9(2.5-3.4)	1.5(1.2-2.0)	296; 4.5(4.0-5.0)	2.5(2.2-2.9)	591; 8.9(8.2-9.7)	1.0(0.9-1.1)	446; 6.7(6.1-7.4)	1.1(1.0-1.3)	356; 5.4(4.8-6.0)	1.3(1.1-1.4)	326; 4.9(4.4-5.5)	0.9(0.8-1.1)	276; 4.2(3.7-4.7)	1.2(1.1-1.2)	187; 2.8(2.4-3.3)	1.0(0.8-1.2)
2014	353; 5.4(4.9-6.0)	1.3(1.2-1.6)	181; 2.8(2.4-3.2)	1.5(1.1-1.9)	257; 3.9(3.5-4.5)	2.3(2.0-2.6)	538; 8.3(7.6-9.0)	0.9(0.8-1.1)	417; 6.4(5.8-7.1)	1.1(1.0-1.2)	358; 5.5(4.9-6.1)	1.3(1.1-1.5)	282; 4.3(3.8-4.9)	0.8(0.7-0.9)	269; 4.1(3.6-4.6)	1.2(1.0-1.3)	183; 2.8(2.4-3.2)	1.0(0.8-1.2)
2015	228; 5(4.4-5.7)	1.2(1.1-1.4)	155; 3.4(2.9-4.0)	1.8(1.4-2.3)	208; 4.5(3.9-5.2)	2.6(2.2-3.0)	462; 9.8(8.9-10.7)	1.1(1.0-1.2)	364; 7.7(6.9-8.5)	1.3(1.2-1.4)	305; 6.5(5.8-7.2)	1.5(1.4-1.7)	217; 4.4(3.9-5.1)	0.8(0.7-0.9)	221; 4.5(3.9-5.2)	1.3(1.2-1.4)	119; 2.4(2.0-2.9)	0.9(0.6-1.2)

a. Incidence per 1000 PYAR
b. Reference year

Supplementary Table 3 n; incidence per 1000 PYAR(95% CI) and IRRs (95%CI) of incident presentations for self-harm by age group and service in males

	10-14 years						15-19 years						20-24 years					
	GP		ED attendances		Hospital admissions		GP		ED attendances		Hospital admissions		GP		ED attendances		Hospital admissions	
	n; inc[a]	IRR	n; inc[a]	IRR	n; inc[a]	IRR	n; inc[a]	IRR	n; inc[a]	IRR	n; inc[a]	IRR	n; inc[a]	IRR	n; inc[a]	IRR	n	IRR
2003	83; 1.1(0.9-1.4)	Ref[b]			30; 0.4(0.3-0.6)	Ref[b]	285; 4.3(3.8-4.9)	Ref[b]			144; 2.2(1.8-2.6)	Ref[b]	248; 4.3(3.8-4.8)	Ref[b]			122; 2.1(1.7-2.5)	Ref[b]
2004	75; 1(0.8-1.2)	0.9(0.7-1.2)			45; 0.6(0.4-0.8)	1.5(1.0-2.0)	296; 4.1(3.7-4.6)	1.0(0.9-1.1)			159; 2.2(1.9-2.6)	1.0(0.9-1.2)	312; 4.7(4.2-5.3)	1.1(0.8-1.5)			162; 2.5(2.1-2.9)	1.2(0.9-1.6)
2005	81; 1(0.8-1.3)	0.9(0.7-1.2)			43; 0.6(0.4-0.7)	1.4(1.0-2.0)	319; 4.3(3.9-4.8)	1.0(0.9-1.1)			168; 2.3(1.9-2.6)	1.0(0.9-1.2)	304; 4.5(4-5)	1.1(0.8-1.4)			144; 2.1(1.8-2.5)	1.0(0.8-1.4)
2006	72; 0.9(0.7-1.2)	0.8(0.7-1.0)			36; 0.5(0.3-0.6)	1.2(0.9-1.5)	342; 4.5(4.1-5)	1.1(1.0-1.2)			194; 2.6(2.2-3)	1.2(1.0-1.3)	293; 4.2(3.7-4.7)	1.0(0.8-1.3)			164; 2.4(2-2.8)	1.2(0.9-1.5)
2007	64; 0.8(0.6-1.1)	0.7(0.6-0.9)			37; 0.5(0.3-0.7)	1.2(0.8-1.8)	351; 4.6(4.1-5.1)	1.1(0.9-1.2)			215; 2.8(2.5-3.2)	1.2(1.1-1.4)	334; 4.7(4.2-5.2)	1.1(0.9-1.4)			204; 2.9(2.5-3.3)	1.4(1.1-1.8)

2008	71; 0.9(0.7-1.2)	0.8(0.6-1.1)			36; 0.5(0.3-0.7)	1.2(0.9-1.6)	327; 4.3(3.8-4.8)	1.0(0.8-1.1)			196; 2.6(2.2-3)	1.1(0.9-1.4)	385; 5.3(4.8-5.9)	1.3(1.0-1.6)			184; 2.6(2.2-2.9)	1.2(1.0-1.6)
2009	62; 0.8(0.6-1.1)	0.7(0.6-1.0)			33; 0.4(0.3-0.6)	1.1(0.7-1.6)	300; 4(3.5-4.5)	0.9(0.8-1.1)			149; 2(1.7-2.3)	0.9(0.8-1.0)	324; 4.4(4-4.9)	1.0(0.8-1.4)			188; 2.6(2.2-3)	1.3(1.0-1.6)
2010	58; 0.8(0.6-1)	0.7(0.6-0.9)			33; 0.4(0.3-0.6)	1.1(0.9-1.4)	372; 5(4.5-5.6)	1.2(1.0-1.3)			160; 2.2(1.8-2.5)	1.0(0.8-1.2)	301; 4.1(3.7-4.6)	1.0(0.7-1.3)			156; 2.1(1.8-2.5)	1.0(0.8-1.3)
2011	55; 0.8(0.6-1)	0.7(0.5-1.0)	45; 0.6(0.4-0.8)	Ref[b]	30; 0.4(0.3-0.6)	1.1(0.7-1.6)	266; 3.7(3.2-4.1)	0.8(0.8-0.9)	299; 4.1(3.7-4.6)	Ref[b]	167; 2.3(2-2.7)	1.0(0.9-1.2)	329; 4.5(4-5)	1.1(0.8-1.4)	313; 4.3(3.8-4.8)	Ref[b]	165; 2.3(1.9-2.6)	1.1(0.8-1.4)
2012	49; 0.7(0.5-0.9)	0.6(0.4-.0.9)	63; 0.9(0.7-1.1)	1.4(1.1-1.9)	35; 0.5(0.3-0.7)	1.3(0.9-1.7)	281; 3.9(3.5-4.4)	0.9(0.8-1.0)	365; 5.1(4.6-5.6)	1.2(1.1-1.4)	148; 2.1(1.7-2.4)	0.9(0.9-1.0)	289; 4(3.5-4.4)	0.9(0.7-1.3)	350; 4.8(4.3-5.3)	1.1(1.0-1.2)	166; 2.3(1.9-2.7)	1.1(0.9-1.5)
2013	69; 1(0.8-1.3)	0.9(0.7-1.2)	78; 1.1(0.9-1.4)	1.8(1.5-2.1)	49; 0.7(0.5-0.9)	1.8(1.2-2.6)	263; 3.7(3.3-4.2)	0.9(0.8-1.0)	307; 4.4(3.9-4.9)	1.1(0.9-1.2)	154; 2.2(1.9-2.6)	1.0(0.9-1.1)	266; 3.7(3.3-4.2)	0.9(0.7-1.2)	313; 4.4(3.9-4.9)	1.0(0.9-1.2)	163; 2.3(1.9-2.7)	1.1(0.8-1.4)
2014	62; 0.9(0.7-1.2)	0.8(0.6-1.0)	69; 1(0.8-1.3)	1.6(1.2-2.3)	39; 0.6(0.4-0.8)	1.4(1.0-2.0)	227; 3.3(2.9-3.8)	0.8(0.6-1.0)	294; 4.3(3.8-4.8)	1.1(0.9-1.2)	140; 2(1.7-2.4)	0.9(0.8-1.0)	264; 3.7(3.3-4.2)	0.9(0.7-1.2)	338; 4.8(4.3-5.3)	1.1(1.1-1.2)	160; 2.3(1.9-2.6)	1.1(0.8-1.5)
2015	44; 0.9(0.7-1.2)	0.8(0.6-1.1)	63; 1.3(1-1.7)	2.1(1.8-2.4)	36; 0.7(0.5-1)	1.9(1.3-2.7)	202; 4(3.5-4.6)	1.0(0.8-1.2)	265; 5.3(4.7-6)	1.3(1.1-1.5)	109; 2.2(1.8-2.6)	1.0(0.8-1.2)	181; 3.4(3-4)	0.8(0.6-1.1)	225; 4.3(3.7-4.9)	1.0(0.9-1.2)	102; 1.9(1.6-2.4)	0.9(0.7-1.2)

a. Incidence per 1000 PYAR
b. Reference year

Supplementary Table 4 IRRs of presentation to services for self-harm by gender

Variable		GP				Emergency Department				Hospital admission			
		Male(n=8506)		Female(n=16345)		Male(n=3387)		Female (n=4161)		Male (n=4665)		Female (n=9304)	
		Incidence (95% CI)	IRR(95% CI)	Incidence (95% CI)	IRR(95% CI)	Incidence (95% CI)	IRR(95% CI)	Incidence (95% CI)	IRR(95% CI)	Incidence (95% CI)	IRR(95% CI)	Incidence (95% CI)	IRR(95% CI)
Age group	10-14	0.9(0.8-1.0)	Reference (P^c<.0001)	4.2(4.1-4.4)	Reference (P^c<.0001)	1.0(0.9-1.1)	Reference (P^c<.0001)	2.6(2.4-2.8)	Reference (P^c<.0001)	0.5(0.5-0.6)	Reference (P^c<.0001)	2.7(2.6-2.9)	Reference (P^c<.0001)
	15-19	4.2(4.0-4.3)	4.6(4.3-5.0)	9.1(8.9-9.3)	2.2(2.0-2.3)	4.6(4.4-4.8)	4.8(4.2-5.4)	6.5(6.2-6.8)	2.5(2.3-2.8)	2.3(2.2-2.4)	4.4(4.0-4.9)	5.0(4.9-5.2)	1.8(1.7-2.2)
	20-24	4.3(4.2-4.4)	4.9(4.5-5.4)	5.5(5.3-5.6)	1.3(1.2-1.4)	4.5(4.3-4.7)	4.8(4.2-5.5)	4.1(3.9-4.3)	1.6(1.4-1.7)	2.3(2.2-2.4)	4.6(4.2-5.2)	2.9(2.8-3.0)	1.0(1.0-1.1)
Deprivation[d]	5	1.5(1.4-1.6)	Reference (P^c<.0001)	8.8(8.5-9.0)	Reference (P^c<.0001)	1.7(1.5-1.8)	Reference (P^c<.0001)	7.8(7.4-8.2)	Reference (P^c<.0001)	0.8(0.7-0.8)	Reference (P^c<.0001)	5.0(4.8-5.1)	Reference (P^c<.0001)
	4	1.9(1.8-2.1)	1.3(1.2-1.5)	6.9(6.7-7.2)	1.2(1.1-1.3)	2.2(2.0-2.5)	1.4(1.2-1.6)	5.8(5.4-6.2)	1.1(1.0-1.3)	1.1(1.0-1.2)	1.5(1.3-1.6)	4.0(3.8-4.2)	1.3(1.2-1.4)
	3	2.6(2.5-2.7)	1.6(1.4-1.7)	5.9(5.7-6.1)	1.4(1.3-1.5)	2.8(2.6-3.0)	1.7(1.5-1.9)	4.1(3.8-4.4)	1.5(1.3-1.7)	1.4(1.3-1.5)	1.6(1.4-1.8)	3.5(3.3-3.6)	1.5(1.4-1.7)
	2	4.1(3.9-4.2)	2.2(2.0-2.4)	5.1(4.9-5.3)	1.7(1.5-1.8)	4.3(4.0-4.6)	2.6(2.3-3.0)	2.7(2.5-3.0)	1.8(1.6-2.0)	2.4(2.3-2.6)	2.5(2.3-2.8)	3.0(2.8-3.1)	1.7(1.6-1.9)
	1	5.7(5.5-5.9)	3.1(2.8-3.3)	4.2(4.0-4.4)	2.1(2.0-2.3)	5.7(5.4-6.0)	3.5(3.1-3.8)	2.4(2.2-2.6)	2.4(2.2-2.7)	3.1(2.9-3.2)	3.2(2.9-3.6)	2.3(2.2-2.4)	2.2(2.2-2.4)
Year	2003	3.1(2.9-3.4)	Reference (P^c<.0001)	6.0(5.7-6.4)	Reference (P^c<.0001)					1.5(1.3-1.7)	Reference (P^c<.0001)	2.9(2.7-3.2)	Reference (P^c<.0001)
	2004	3.2(2.9-3.4)	1.0(0.9-1.2)	6.1(5.8-6.5)	1.0(0.9-1.1)					1.7(1.5-1.9)	1.1(0.9-1.3)	2.9(2.6-3.1)	1.0(0.8-1.1)
	2005	3.2(3.0-3.5)	1.0(0.9-1.2)	6.2(5.9-6.6)	1.0(1.0-1.1)					1.6(1.5-1.8)	1.1(0.9-1.2)	3.4(3.2-3.7)	1.2(1.0-1.4)
	2006	3.2(3.0-3.4)	1.0(0.9-1.1)	6.1(5.8-6.5)	1.0(0.9-1.1)					1.8(1.6-2.0)	1.2(1.0-1.3)	3.6(3.4-3.9)	1.2(1.1-1.4)
	2007	3.3(3.1-3.6)	1.0(0.9-1.2)	6.8(6.5-7.2)	1.1(1.0-1.2)					2.0(1.9-2.2)	1.3(1.1-1.5)	3.9(3.7-4.2)	1.3(1.2-1.5)

Surgical ED Surgical Paediatric Psychiatric General Other

2008	3.5(3.2-3.7)	1.1(0.9-1.3)	6.8(6.5-7.2)	1.1(1.0-1.2)					1.9(1.7-2.0)	1.2(1.0-1.4)	3.8(3.5-4.0)	1.3(1.1-1.5)
2009	3.1(2.8-3.3)	0.9(0.8-1.1)	5.9(5.5-6.2)	1.0(0.9-1.1)					1.7(1.5-1.8)	1.1(0.9-1.3)	3.2(3.0-3.5)	1.1(1.0-1.3)
2010	3.3(3.1-3.6)	1.0(0.9-1.2)	6.0(5.7-6.3)	1.0(0.9-1.1)					1.6(1.4-1.8)	1.0(0.9-1.2)	3.1(2.9-3.4)	1.1(0.9-1.2)
2011	3.0(2.8-3.2)	0.9(0.8-1.0)	5.9(5.5-6.2)	1.0(0.9-1.1)	3.0(2.8-3.2)	Reference (P[c]<.0001)	3.8(3.6-4.1)	Reference (P[c]<.0001)	1.7(1.5-1.8)	1.1(0.9-1.2)	3.4(3.2-3.7)	1.2(1.0-1.3)
2012	2.9(2.6-3.1)	0.9(0.8-1.0)	6.3(5.9-6.6)	1.0(0.9-1.1)	3.6(3.4-3.9)	1.2(1.1-1.3)	4.2(3.9-4.5)	1.1(1.0-1.2)	1.6(1.5-1.8)	1.0(0.9-1.2)	3.5(3.2-3.7)	1.2(1.0-1.4)
2013	2.8(2.6-3.1)	0.9(0.8-1.0)	6.8(6.4-7.1)	1.1(1.0-1.3)	3.3(3.1-3.6)	1.1(1.0-1.2)	4.6(4.3-4.9)	1.2(1.1-1.3)	1.7(1.6-1.9)	1.1(0.9-1.3)	4.2(3.9-4.5)	1.5(1.2-1.7)
2014	2.7(2.4-2.9)	0.8(0.7-1.0)	6.0(5.7-6.4)	1.0(0.9-1.1)	3.4(3.1-3.6)	1.1(1.0-1.2)	4.4(4.1-4.7)	1.2(1.1-1.3)	1.6(1.5-1.8)	1.1(0.9-1.2)	4.1(3.8-4.4)	1.4(1.2-1.6)
2015[e]	2.8(2.6-3.1)	0.9(0.7-1.0)	6.4(6.0-6.8)	1.0(0.9-1.2)	3.7(3.4-4.0)	1.2(1.1-1.4)	5.2(4.8-5.6)	1.4(1.2-1.5)	1.6(1.4-1.8)	1.0(0.9-1.2)	4.5(4.1-4.8)	1.5(1.3-1.8)

a. Emergency department data from 2011 onwards only
b. Adjusted for calendar year, age and deprivation
c. Based on Wald test
d. Deprivation: 1 = most deprived; 5 = least deprived
e. Data collected in 2015 up until 30th September –denominator for incidence rate adjusted accordingly

Supplementary Table 5 n(%;95% CI) of hospital admissions for self-harm by admission speciality, age group and gender

		Specialties	Specialty			Medicine	
Age group	10-14 years	23(1;1-1)	<5	2740(99;98-99)	<5	<5	<5
	15-19 years	74(3;3-3)	<>(16;15-18)	<>(30;28-31)	<>(1;1-1)	<>(36;34-38)	<>(13;12-15)
	20-24 years	217(6;5-7)	813(22;20-23)	<5	47(1;1-2)	2035(54;53-56)	642(17;16-18)
Sex	Male	186(7;6-8)	449(17;16-19)	502(19;18-21)	32(1;1-2)	1077(41;40-43)	353(14;12-15)
	Female	128(2;2-3)	717(12;11-13)	2870(47;46-49)	37(1;0-1)	1736(29;27-30)	578(10;9-10)
**All (n=8665)**		**314(4;3-4)**	**1166(13;13-14)**	**3372(39;38-40)**	**69(1;1-1)**	**2813(33;32-33)**	**931(11;10-11)**

a. Encompasses all other specialties

<>. Numbers excluded to make small numbers

Supplementary Table 6 Proportion of ED attendances associated with hospital admission by method gender and age group 01.08.2009-30.09.2015

		All self-harm		Self-poisoning		Self-injury		Both Self-poisoning and injury	
		ED attendances (n)	With hospital admission (n (%; 95% CI)	ED attendances (n)	With hospital admission (n (%; 95% CI)	ED attendances (n)	With hospital admission (n (%; 95% CI)	ED attendances (n)	With hospital admission (n (%; 95% CI)
Male		5059	1751(35;33-36)	1065	468(44;41-47)	3317	1048(32;30-33)	677	235(35;31-38)
Female		6780	3104(46;45-47)	1670	936(56;54-58)	4031	1655(41;40-43)	1079	513(48;45-51)
10-15 years	All	2108	1456(69;67-71)	517	448(87;83-89)	1252	741(59;56-62)	339	267(79;74-83)
	Male	560	274(49;45-53)	91	63(69;59-78)	386	359(93;90-95)	83	49(59;48-69)
	Female	1548	1182(76;74-78)	426	385(90;87-93)	866	700(81;78-83)	256	218(85;80-89)
16-18 years	All	3444	1239(36;34-38)	751	349(46;43-50)	2166	719(33;31-35)	527	171(32;29-37)
	Male	1319	382(29;27-31)	263	106(40;35-46)	888	242(27;24-30)	168	34(20;15-27)

	Female	2125	857(40;38-42)	488	243(50;45-54)	1278	477(37;35-40)	359	137(38;33-43)
19-24 years	All	6287	2160(34;33-36)	1467	607(41;39-44)	3930	1243(32;30-33)	890	310(35;32-38)
	Male	3180	1095(34;33-36)	711	299(42;38-46)	2043	644(32;30-34)	426	152(36;31-40)
	Female	3107	1065(34;33-36)	756	308(41;37-44)	1887	599(32;30-34)	464	158(34;30-38)
**Total**		**11839**	**4855(41;40-42)**	**2735**	**1404(51;49-53)**	**7348**	**2703(37;36-38)**	**1756**	**748(43;40-45)**
